# Bi-Parental Care Contributes to Sexually Dimorphic Neural Cell Genesis in the Adult Mammalian Brain

**DOI:** 10.1371/journal.pone.0062701

**Published:** 2013-05-01

**Authors:** Gloria K. Mak, Michael C. Antle, Richard H. Dyck, Samuel Weiss

**Affiliations:** 1 Hotchkiss Brain Institute, Department of Cell Biology & Anatomy, Faculty of Medicine, University of Calgary, Calgary, Alberta, Canada; 2 Hotchkiss Brain Institute, Department of Physiology and Pharmacology, Faculty of Medicine, University of Calgary, Calgary, Alberta, Canada; 3 Hotchkiss Brain Institute, Department of Psychology, Faculty of Arts, University of Calgary, Calgary, Alberta, Canada; University of Victoria, Canada

## Abstract

Early life events can modulate brain development to produce persistent physiological and behavioural phenotypes that are transmissible across generations. However, whether neural precursor cells are altered by early life events, to produce persistent and transmissible behavioural changes, is unknown. Here, we show that bi-parental care, in early life, increases neural cell genesis in the adult rodent brain in a sexually dimorphic manner. Bi-parentally raised male mice display enhanced adult dentate gyrus neurogenesis, which improves hippocampal neurogenesis-dependent learning and memory. Female mice display enhanced adult white matter oligodendrocyte production, which increases proficiency in bilateral motor coordination and preference for social investigation. Surprisingly, single parent-raised male and female offspring, whose fathers and mothers received bi-parental care, respectively, display a similar enhancement in adult neural cell genesis and phenotypic behaviour. Therefore, neural plasticity and behavioural effects due to bi-parental care persist throughout life and are transmitted to the next generation.

## Introduction

Early life experiences have a profound effect on brain development, emotionality, and social behaviours throughout life [1], [2]. Adverse prenatal and postnatal events can affect cognition and alter the hypothalamic -pituitary, -adrenal and -gonadal axes to modulate stress reactivity and reproductive behaviours differently in adult males and females [3], [4], [5]. Conversely, enriched early life experiences, such as increased maternal care [4] or communal nesting [1], tend to decrease stress and emotional reactivity, as well as provide enhanced cognitive benefits. Similarly, neonatal exposure to novelty improves social recognition and spatial memory in adulthood [6], [7]. Such enriched early life experiences can also have differential effects in males and females [8], [9].

Plasticity changes in the hippocampus have been implicated in mediating many behavioural outcomes associated with early life experiences. Low maternal care leads to decreased hippocampal synaptic plasticity [10] and glucocorticoid receptor expression, which results in heightened stress reactivity [4]. Interestingly, decreased maternal care in humans and prenatal stress in rodents is associated with decreased hippocampal volume [11] and decreased numbers of hippocampal neurons, respectively, in females, but not males [12], [13]. Therefore, social stress during early postnatal development has sex-specific effects on brain development and adult behaviour [14]. Recently, it has been demonstrated that double-mothering enhances adult male hippocampal dendritic plasticity and performance on hippocampal-dependent tasks. However, the effect double-mothering has on hippocampal plasticity and function in adult females is unknown [15]. Therefore, little is known as to whether enriched early life environments give rise to sexual dimorphic brain plasticity and behavioural outcomes.

Remarkably, the behavioural phenotypes arising from early life adversities [2], [16], [17] and synaptic physiological phenotypes from enriched environments [18] are transmissible across generations. However, little is still known about whether brain plasticity and cognitive enhancements associated with enriched or novel early life experiences are transmitted to subsequent generations. Therefore, insight into the long term intergenerational consequences of early life experiences, coupled with how such experiences may differentially impact brain plasticity and function in males and females, may be important for understanding the effects of developmental origins of health and disease.

Given that early life adversities have a life-long impact on adult brain plasticity and social behaviours, perhaps lifelong cell genesis in the brain is also influenced by early life experiences. In the adult mammalian brain, neural cell genesis persists in the subventricular zone (SVZ) [19], subgranular zone (SGZ) [20], and subcallosal zone (SCZ) [21]. Newly generated neurons produced from neural stem cells in the SVZ and SGZ give rise to new neurons in the olfactory bulb and dentate gyrus of the hippocampus, respectively [19], [20]. Whereas, the neural stem cell populations in the SVZ and SCZ contribute to new myelinating oligodendrocytes in the corpus callosum [21], [22]. Maternal separation in early life has been shown to decrease neurogenesis in the dentate gyrus, but not the olfactory bulb [23]. Moreover, increased parental care, through communal nesting or enhanced maternal care [24], [25], and postnatal environmental enrichment [26] increases the survival of new neurons in the dentate gyrus. Therefore we asked whether parental care, administered by two individuals (bi-parental care), modulates adult neural cell genesis in males and females to give rise to specific behavioural phenotypes. Further, given the phenotypical transmissibility associated with early life events [2], [16], [17], [18], we also asked whether such changes in adult neural cell genesis and their resulting behavioural correlates, are transmissible to the next generation.

## Materials and Methods

### Ethics Statement

All procedures were approved by the University of Calgary Animal Care Committee (protocol numbers: M08110, M10032, M10033, M06105) and conformed to the guidelines outlined by the Canadian Council on Animal Care.

### Animals and Parental Environment Conditions

We used 8-week old female and male mice throughout this study. C57BL6 breeding pairs were obtained from Charles River (Laval). Animals were handled in accordance with the animal care policies of the University of Calgary. Animals were maintained on a 12hour light/dark cycle with food and water *ad libidum*. All mice were provided with nestlets and Plexiglas igloo-style houses as part of the standard enrichment at the University of Calgary animal facility. Female and male mice were mated, where mating was detected by the presence of a plug. Pregnant females were placed into different conditions so that pups would experience different parental conditions until weaning (21 days of age). It has been suggested that mice are particularly sensitive to external disturbances, which increases the likelihood of individual mouse pup and whole litter mortality [27]. Given the high pup mortality rate reported for C57BL6 mice [28], pups born in the different parental conditions were left undisturbed to prevent mortality. As a result, pups were not culled nor were the sex ratios of the litter equalized, as this would involve physical displacement of pups and the nest, leading to disturbance of the cage environment, which has been shown to influence pup mortality [29], [30].

Maternal only condition: Impregnated females were separated into individual cages. After 18 days, females gave birth to a litter of 6–8 pups and raised the pups on their own.

Maternal-virgin condition: Impregnated females were placed into individual cages with a virgin age-matched female and would remain together for duration of pregnancy (18 days). After 18 days, the pregnant females would give birth to a litter of 6–8 pups. The pair would remain together with the pups until weaning.

Maternal-paternal condition: Pairs of male and female mice were placed in individual cages. Mating was detected by the presence of a plug and males remained with their partners for the duration of pregnancy and with the pups until weaning.

After the animals were weaned from their specific rearing environments (at postnatal day 21), they were socially housed with littermates. A total of 269 animals were used in this study. The actual numbers of animals used per analyses are detailed in the figure legends.

### Animal Behavioural Tasks

A behavioural test battery, ordered from least to most stressful, was conducted [31], [32]. While the advantages of using a behavioural test battery are many, there is the possibility of mice exhibiting training effects, where one test influences another [31]. As such, we performed a series of tasks that were in similar order outlined in McIlwain et al [31], keeping in mind that some tests may be more sensitive to previous testing experience. Open field was first administered, followed by light-dark, elevated plus maze, Y-Maze, ladder rung, social preference, pre-pulse inhibition, Morris water maze, passive avoidance, and cue/contextual fear conditioning. The duration between conducting the first five tasks was 24 hours. After the social preference task, animals were left undisturbed for 1 week followed by the administration of pre-pulse inhibition. A one-week inter-test duration was employed between the pre-pulse inhibition, Morris Water Maze, passive avoidance and cue/contextual fear conditioning tasks. Naive mice from second generation breeding (i.e. offspring from MO, MV and MP parents) were specifically tested on behavioural assays that first generation biparentally raised mice demonstrated significant differences in. Performance on behavioural tasks, such as cue/contextual fear conditioning, have been demonstrated not to be dependent on previous training [31].

Some studies using rats have demonstrated that performance on spatial memory tasks are independent of estrus cycle phase [33], [34], whereas others have described impaired spatial memory during the proestrus and estrus phase [35], [36], where estrogen is at its peak. In mice, the administration of estradiol has conflicting results on whether it improves or impairs spatial memory [37], [38]. Moreover, a study by Frick et al demonstrated that mice use very different strategies than rats in the Morris water maze [39]. Given that all mice are grouped housed from weaning until behavioural testing, it is possible that these grouped housed mice experience anoestrus [40]. Further Lagace et al demonstrate that hippocampal neurogenesis is not influenced by the estrous cycle in C57BL6 mice and suggest that their data supports the use of female mice without determining estrous cycle stage [41]. As such, female mice underwent behavioural testing without taking into account individual estrous cycle phase.

A surgical procedure was necessary to carry out telemetric measures. Therefore, telemetry was monitored in separate groups of animals that did not undergo the behavioural test battery. Home cage activity was telemetrically monitored in mice as described in Antle et al [42]. Male mice were surgically implanted with an intraperitoneal transmitter. Animals were allowed 7 days to recover from the surgery in individual cages before testing. Locomotor activity was assessed by frequencies emitted by the implanted transmitters. Frequencies were collected by individual receivers placed under each cage for 1 week. Data from the receivers were sent to a separated computer equipped with ER-4000 Energizer/Receivers and running Vital View data collection software (Minimitter, Bend, OR). Locomotor activity was recorded across the entire week of observation and averaged to obtain mean values for 10 minute intervals.

Open field analysis was conducted using the methods outlined in Sgado et al [43]. Briefly, open field behaviour was recorded and quantified using an automated video tracking system. The open field arena was circular (1.55 m diameter, 0.46 m height) constructed of white fiberglass. Mice were permitted to freely explore for 5 minutes while an image tracking system (HVS Image, Hampton, UK) recorded total distance traveled, average walking speed, and percent time spent in the core, middle, and periphery of the arena. The arena was cleaned between trials with 70% ethanol.

The Y-maze task was conducted based on methods described in Saxe et al [44]. Briefly, the Y-maze was constructed out of three Plexiglas arms of equal size joined together in a Y configuration. Each arm was 40 cm long and 10 cm wide with 12 cm high walls. The floor of each arm was white and the walls were clear. The apparatus was placed at ground level and all sessions were video captured for subsequent analysis. Individually, mice were allowed to explore two out of the three arms for a total of 10 minutes. The third arm was block with a piece of Plexiglas that could be slid in and out. Thirty minutes later the mice were individually returned to the maze for 5 minutes with all arms open. The number of entries and time spent in each arm were quantified.

The elevated plus maze was conducted based on methods previously described in Malleret et al [45]. Briefly, the plus maze consisted of four black Plexiglas arms, two open arms (67 cm×7 cm) and two enclosed arms (67 cm×7 cm×17 cm) that forms a cross shape with the open arms opposite of each other. The maze was elevated 55 cm above ground. Animals were placed in the center of the maze and explored for 10 minutes. A camera was mounted directly above the maze to record the behaviour of the animal. The number of entries into the open versus enclosed arms were counted, as well as the time spent in the open versus enclosed arms. An anxiety ratio was calculated for each animal by dividing the amount of time spent in the open arms by the total time in the open plus enclosed arms.

The light-dark task was based on methods described in Saxe et al [44]. Briefly, the light-dark task was conducted in a Plexiglas square arena measuring 44 cm×44 cm with white floors and clear walls. A dark tinted Plexiglas cover was created to fit over half of the arena. An opening at the floor level in the centre of the tinted Plexiglas cover allowed for passage between the light and dark compartments. The light compartment was brightly illuminated with a fluorescent light. Mice would be placed in the dark compartment to begin with and allowed to freely explore both compartments for 5 minutes, while being video recorded. Time spent in each of the compartments was quantified.

The horizontal ladder rung consists of clear Plexiglas walls and removable metal rungs (3 mm diameter) with a minimum distance of 1 cm between each rung. The rung walkway is 1 meter in length, the walls span 1 m and measures 19 cm high from the height of the rungs. The entire ladder is elevated 30 cm above the ground, the width of the 1 meter walkway is 6 cm to prevent the mouse from turning around with ease. During training and testing, the home cage was placed at one end of the rung. Animals were trained over several sessions with rungs all separated by a 1 cm distance until they were able to walk the length of the rung with speed and without hesitation. The difficulty of the task was modified by varying the position of the rungs by removing rungs along a 40 cm portion midway along the length of the walkway. An irregular pattern of spacing the rungs was changed each trial session to trial session to prevent animals from learning the pattern. The distance of the rungs varied from 1 cm to 3 cm for the irregular patterns. Trial sessions consisted of 5 complete walks along the length of the apparatus. Between trials the apparatus was cleaned with 70% ethanol. A video camera recorded paw placement on the rungs. Evaluation of forelimb and hindlimb paw placement was assessed using a modified version presented in Metz and Whishaw [46]. The total number of steps taken within the 40 cm portion was quantified in addition to the number of full slips, which were given a score of 1 and half-slips, which were given a score of 0.5. A full slip would entail the animal’s entire paw and portion of leg falling in the space between two metal rungs, thus breaking the rhythmic stride of the animal. Whereas a half-slip would entail only the animal’s placement of a paw between the space of two metal rungs, thus its rhythmic stride is maintained. A slip ratio was then calculated by taking the score obtained in a session divided by the number of steps taken to cross the 40 cm portion of irregularly spaced rungs. Slip ratios for each of the five trials were then averaged.

The social preference of mice was tested based on methods described in Fairless et al with slight modifications [47]. A Plexiglas chamber that could be compartmentalized into three compartments by perforated dividers was constructed. The perforations were large enough to allow for the exchange of visual, auditory, odor, and tactile (part of head only) information. Animals were habituated to the chamber for 5 minutes each day, two days prior to testing. The lack of preference for either side of the chamber was determined by a 10 minute exploratory session post-habituation. To test preference, mice were placed in the center of the chamber and a novel same-sex conspecific or inanimate object (clear plastic cube) was placed on either end of the chamber. Animals were video recorded for 10 minutes, the duration of time spent investigating each side was quantified.

Prepulse inhibition was conducted in an apparatus from SR-LAB, San Diego Instruments, San Diego, CA, USA, which was connected to a computer to monitor the startle response of individual animals placed in the apparatus. Animals were acclimatized to the apparatus for 3 minute with 65 dB of noise. A habituation phase served to stabilize the animal’s startle response, which consisted of ten pulse trials (40 ms, 120 dB). Subsequently, ten blocks of trials were presented. Each block consisted of a) ten pulse-alone trials (40 ms, 120 dB); b) ten pre-pulse plus pulse trials (pre-pulse: 20 ms, 80 dB; pulse: 40ms, 120 dB) c) ten no pulse trials (40 ms, 65 dB). The 30 trials were presented in a pseudorandom order within each block, with a variable intertrial interval of a mean duration of 15 seconds. Percent pre-pulse inhibition was analyzed by converting the startle data into percent scores: (% PPI = 100%×(pulse alone – prepulse plus pulse)/pulse alone).

Behaviour in the water maze was recorded and quantified using an automated video tracking system. The water maze consisted of a circular swimming pool 1.55 m in diameter and 0.46 m in height, constructed of white fiberglass. The pool was located in a room with various spatial cues. The pool was filled with 18 C water (30 cm in depth) and made opaque with skim milk. A platform, serving as a refuge from the water, was located in an arbitrarily defined quadrant of the maze, and 2 cm below the surface of the water so that the mice could not see the platform when swimming. Mice were recorded from a video camera directly mounted above the pool and connected to a computer with an image tracking system (HVS Image, Hampton, UK) which recorded total distance taken to get to the hidden platform, latency to reach the platform, average swimming speed, percent time spent in each quadrant. The water maze task was conducted based on methods described in Saxe et al [44]. On the first day, mice had AM and PM training sessions separated by 4 hours. Each session consisted of four-trials, where mice were released from the north, east, south, and west points of the pool with the platform in the center of an arbitrarily defined quadrant. Between each release points, mice were placed back in their home cage for a 10 minute inter-trial period. In each of the trials, mice were given 60 seconds to locate the platform, once found, they would remain on the platform for 10 seconds and this would end the trial. Mice unable to find the platform within the 60 seconds were placed on the platform for 10 seconds, thus ending the trial. On the second and third day, the mice would repeat the trials as described, with the platform remaining in the same location, while varying the sequence of release. After three days of training, animals were given a probe trial. The platform would not be placed in the pool during the probe trial and the mice would swim for the entire 60 seconds in duration, then removed from the pool. To assess long-term spatial memory, mice would be tested again in the same manner as the probe trial 7 days later. Data from the tracking software was converted into Microsoft Excel format to allow further data analysis.

For passive avoidance, training and retention trials were conducted in a chamber which had internal dimensions of approximately 45 cm×15 cm×25 cm (Hamilton-Kinder, LLC, San Diego, CA). Mice were placed in one half of the chamber and allowed to habituate for 2 minutes, after the 2 minutes a gate would open, allowing the mouse access to the other side of the chamber. When the mouse stepped onto the metal rungs on the other side of the chamber it would receive a foot shock (0.7 mA for 1.5 seconds). The chamber is connected to a computer that measures the latency to cross from the side the mouse was originally placed to the opposite side. The chamber was cleaned with 70% ethanol between each session. 48 hours and 7 days after the initial training session, animals were tested for retention using the same procedure as described, however no shock was administered. The latency to cross to the other side was measured by the computer, with a maximum allotted time of 300 s to cross.

Fear conditioning was conducted based on methods outlined in Saxe et al [44]. Briefly, fear conditioning was conducted in a chamber with removable plastic walls and a metal rung floor 3 cm above the base of the apparatus (Hamilton-Kinder, LLC, San Diego, CA). The internal dimensions of the chamber were approximately 20 cm×15 cm×25 cm. The chamber was located inside a larger, insulated metal and plastic cabinet which provided protection from outside light and noise. The behaviour of the mice was recorded by a digital video camera directly mounted above the conditioning chamber. Amount of time spent freezing was quantified, defined by the complete absence of motion.

Fear conditioning was conducted over three days. Day one, mice were placed in the conditioning chamber with black walls and received three pairings between a tone (20 s, 80 dB) and a coterminating shock (1 s, 0.5 mA). The inter-trial interval between each of the pairings was 2 minutes. Mice were habituated to the chamber for 2 minutes, after which the first tone-shock pairing was presented. The chamber was cleaned with 70% ethanol after each mouse. On the second day, the procedure and context were changed in several ways to test conditioned fear of the tone in the absence of contextual cues associated with the shock. The walls were made white; the chamber was scented with coconut extract; the experimenter wore different material gloves; a non-alcohol disinfected was used; and mice were kept in a different room before testing. Each mouse was placed in the chamber for approximately 6 minutes. A tone was presented twice, 120 seconds, and 260 seconds after being placed into the chamber. No shocks were administered. Freezing was scored 1 minute before the first presented tone (pre-tone) and during the 20 s of the first tone (tone). On day three, mice were tested for conditioned fear in the original training context. The testing procedure and context were identical to those used on day one, except that the shock was not presented. Each minute within the entire session was scored for freezing.

### Parental Observations

Males and females in the three different parental environment conditions were left undisturbed during observations. Four litters were observed per parental environment condition. The four litters in which parental observations were performed per parental environment condition contributed to a subset of animals to the study. Additional males and females were used to generate the numbers of animals required to carry out the study to eliminate litter specific effects. The duration of time spent conducting the parental behaviours of: arch back nursing (female is in arched position over pups), licking and grooming, nest building, resting in nest and off nest, was recorded by an observer. Parental behaviours were observed once per minute in 15 minute increments four times each day (08∶00, 10∶30, 14∶00, 17∶30, with two observations during the light cycle and the other two during the dark cycle with red light illumination) from P0-P10. Visible ear notches were used to identify the virgin females and males in the maternal-virgin and maternal-paternal conditions, maternal females did not have their ears notched.

### BrdU Administration

Mice were given BrdU (Sigma) (120 mg/kg, i.p. dissolved in 0.007% NaOH in phosphate buffer) every 2 hours for 10 hours and killed 16 hours after the final injection. Mice were given 10 hours of BrdU to label the entire constitutively proliferating population of cells in the subventricular zone, as previously outlined in Morshead et al [48]. To detect the phenotype of BrdU labeled cells in the dentate gyrus and corpus callosum BrdU was administered at the appropriate time points, then animals were allowed to survive for an additional 3–4 weeks or 12 days, if analyzing the dentate gyrus and corpus callosum, respectively. Animals would then be killed and tissue processed for immunohistochemistry analysis as outlined below.

### Tissue Histology

These methods are detailed in previous studies [49], [50]. The animals were killed by anesthetic overdose of Somnotol and perfused transcardially with 4% paraformaldehyde in PBS, pH 7.2. Brains were post-fixed in the same paraformaldehyde solution overnight at 4°C, and cyroprotected for 24 hours in 20% sucrose in PBS. The brains were then embedded in Tissue Tek O.C.T. compound (Sakura Fineteck, Torrance, CA) before coronal sections of the subventricular zone, hippocampus, and spinal cord were cyrosectioned at 14 µm. Before immunocytochemistry, the sections were post-fixed with acetone for 30 seconds at room temperature and washed with PBS. For BrdU staining, the tissue was treated with 1 M HCl for 22 minutes at 60°C to denature the cellular DNA. Rat monoclonal anti-BrdU (1∶200, Oxford Biotechnology, Oxfordshire, UK) and rabbit polyclonal anti-Ki67 (1∶500, Novacastra, Newcastle upon Tyne, UK) were used to detect proliferating cells in the dentate gyrus. Sections were then incubated overnight at room temperature in primary antibody diluted in 0.3% PBS-T containing 10% NGS, washed with PBS, and then incubated with donkey or goat biotinylated secondary antibodies (all used at 1∶200, Jackson ImmunoResearch, Westgrove, PA, USA) for 1 hour at room temperature. This was followed with 45 min incubation at room temperature with streptavidin-Cy3 (1∶1500, Jackson ImmunoResearch) and Hoechst 33258 (0.015 mg/ml stock solution diluted to 0.001 mg/ml, Sigma) in PBS. After rinsing with ddH_2_O, sections were mounted with Fluorosave (Calbiochem, San Diego, CA) and viewed with a Zeiss Axiophot fluorescence microscope. For double labeling in the corpus callosum, and dentate gyrus, sections were first processed for BrdU immunocytochemistry, using donkey anti-rat IgG conjugated to fluorescein as the secondary antibody. Subsequently, slides were processed with NeuN (1∶200; Santa Cruz Biotechnology, Santa Cruz, CA); platelet-derived growth factor receptor alpha (PDGFRa) (1∶10; R & D Systems, Minneapolis, MN), or for detection of glutathione S-transferase-π (GSTπ), sections were washed in PBS, then placed in boiling 0.01 M citrate buffer, pH 6.0 for 1.5 minutes prior to application of any antibody. Mouse anti-GSTπ (1∶50; BD Biosciences PharMingen, San Diego, CA) was used.

To detect cell death using TUNEL staining, slides were re-hydrated in PBS for 10 minutes and then incubated in 3% hydrogen peroxide in methanol for 10 minutes at room temperature. Sections were washed in PBS and treated with 20 µg/µl proteinase K diluted 1∶1000 in 10 mM Tris-HCl (pH 8) for 2.5 minutes at room temperature and washed with PBS. Sections were treated with 100 µl of TUNEL reaction mixture (ROCHE In Situ Cell Death Kit Fluorscien (FITC)), as outlined by the manufacturer and incubated for 1.5 hrs at 37°C. Subsequent immuno-detection was done with anti-FITC (1∶400, Sigma) overnight at 4°C, followed by biotinylated goat anti-mouse (1∶200), streptavidin-FITC (1∶1000) (Jackson Immunoresearch) and Hoechst 33258 (1∶100).

### Cell Quantification

For the subventricular zone, a one-in-ten series of coronal sections (14 µm) from the rostral tip of the lateral ventricle to 1400 µm caudal of the ventricles were collected. Cells labeled by both the antibody of interest and Hoechst 33258 staining were counted blind in the defining SVZ. Cells labeled with the antibody of interest, but not with Hoechst 33258 and cells not clearly labeled with the antibody of interest were not counted.

For the hippocampus, a one-in-ten series of every other coronal section (14 µm) from the rostral portion of the hippocampus to 2800 µm caudal was collected. Cells were counted blind in the dentate gyrus as defined by Hoechst 33258 staining. Cells labeled with the antibody of interest, but without Hoechst 33258 and cells not clearly labeled with the antibody of interest were not counted. In the case of BrdU/NeuN double labeled cells, cell counts were performed blind and only cells clearly labeled with BrdU and NeuN were counted. Cells within the dentate gyrus of each representative slide of an entire hippocampus (10 sections) were counted using confocal microscopy under a 40x objective with Olympus Microsuite tissue analysis software. The software created and complied the Z-stacks for labeled cells within the dentate gyrus so that all distinctly labeled cells would be counted while labeled artifacts were discounted. Stereology was conducted using StereoInvestigator software (Microbrightfield Bioscience) with 10 coronal sections immuno-stained with anti-BrdU. The entire dentate gyrus area was quantified by tracing the perimeter using a 2.5x objective in each of the coronal sections, the software calculated the total area. BrdU-labeled cells were counted using the StereoInvestigator software with the same tracings for the area measurements. BrdU-labeled cells were counted in a 50 µm×50 µm counting frame with a 100 µm×100 µm grid size.

For the corpus callosum, a two-in-ten series of coronal sections (14 µm) from the rostral tip of the corpus callosum to 2800 µm caudal was collected. BrdU, BrdU/PDGFRa, or BrdU/GSTπ -positive cells were counted from the lateral-medial regions of the corpus callosum blind, in both hemispheres. Only cells that were clearly BrdU, PDGFRa, GSTπ, and Hoeschst 33258 labeled were included in the quantification.

For the spinal cord, a two-in-eight series of coronal sections (14 µm) were cut from cervical, lumbar, and thoracic regions of the spinal cord. The representative regions of the spinal cord were pre-cut prior to embedding the tissue in OCT and sectioning. BrdU positively labeled cells, which co-labeled with Hoeschst 33258 were included in the quantification.

### Imaging

All fluorescent images were acquired on a Zeiss Axiophot fluorescence microscope using 25×, 40×, and/or 63× objectives, with the Rhodamine, FITC, and/or DAPI filters. Images were obtained using the AxioVision 3.1 software program at a resolution of 2600×2060 in the basic adjustment RGB mode. Images were saved as TIFF files and then transferred into Adobe Photoshop7.0 for processing. Adobe Photoshop7.0 manipulations involved cropping and varying the brightness and contrast, where the latter was applied equally across the entire image.

### Statistical Analysis

Data were analyzed statistically using GraphPad Prism (San Diego, CA) or SPSS (Armonk, NY). Analysis of significant differences was performed using a two-tailed student’s t-test when an experiment only had two groups to compare or one- or two- way ANOVA followed by Tukey’s post-hoc analysis for experiments with more than two groups, or repeated measures ANOVA when similar data was collected from groups of animals over a number of days. P-value was set at 0.05.

### Gross Brain Myelin Staining, Electron Microscopy and Quantification

A one-in-six series (14 µm) of sagittal sections starting medial and advancing 112 µm lateral was collected. Eriochrome cyanine staining was performed to visualize myelin, whereas the gray matter was counterstained with Eosin. This staining procedure is outlined by Kiernan [51]. Briefly, slides were placed in the staining solution (0.21 M aqueous ferric chloride (Sigma-Aldrich), 1.0 g Eriochrome cyanine R (Sigma-Aldrich), 2.5 ml sulphuric acid (Sigma-Aldrich), made up to 500 ml with dH_2_O). The intensity of staining was monitored periodically, after which slides were placed in dH_2_O and differentiated in 0.21 M aqueous ferric chloride. After differentiation, the gray matter was counterstained in Eosin then rinsed in dH_2_O. Slides were dehydrated in ethanol, cleared with xylene, and then mounted with mounting medium. Slides were viewed with a light microscope, digital images of the region of interest were taken and the area of corpus callosum present within the region of analysis was assessed with ImageJ software.

### Electron Microscopy

Female mice were perfused with saline containing 100 IU/ml heparin, followed by 10 minutes of 3% glutaraldehyde in Sorenson’s buffer. Brains were extracted from the animals and cut down the midline, then post-fixed for 1 hour in 3% glutaraldehyde in Sorenson’s buffer. Sections were then cut at 80 µm and the region of analysis (genu of corpus callosum) was dissected out (∼1 mm^3^ in size). The tissue was placed in 2.5% glutaraldehyde in 0.1 M cacodylate buffer overnight at 4C. The tissue was then transferred into a new vial containing 0.1 M cacodylate buffer and washed 3 times. Tissue was left in 0.1 M cacodylate buffer overnight at 4C and subsequently placed in 1% osmium tetroxide/0.1 M cacodylate buffer for ∼1 hr with gentle rotation. Tissue was then rinse with ddH_2_O, serially dehydrated in ethanol, and subsequently serially infiltrated with Epon resin. Tissue was then placed in a capsule with Epon resin and polymerize at 60C for 24 hours. Ultrathin sections were collected on grids and subsequently processed with 5% uranyl acetate solution and Reynolds lead citrate solution prior to imaging. All solutions used for electron microscopy analysis were obtained from Electron Microscopy Sciences, USA. Fifteen photographs were randomly taken from each genu of corpus callosum at 5000×. The number of myelinated axons per field was counted based on methods previously outlined in Gregg et al [49].

## Results

To investigate the effects bi-parental care may have on adult neural cell genesis, females were mated with male mice. Pregnant females were then placed into: individual cages to raise their pups alone (Maternal-only condition); placed into individual cages with a virgin female to help raise pups (Maternal-virgin condition); left with the male they mated with to help raise pups (Maternal-paternal condition). From these parental environment conditions, we conducted the following experimental paradigm outlined in Figure S1. By conducting parental care observations, four times a day from postnatal day (P) 0 to P10, we found that females in the three different parental conditions displayed no difference in the duration spent arch back nursing, licking and grooming, nest building, resting in the nest, and off nest (Fig. S2A–E). Interestingly, the virgin females and males within the maternal-virgin and maternal-paternal conditions did not display differences in the duration spent licking and grooming, nest building, resting in the nest, and off nest (Fig. S3A–D). When we totaled and averaged the time spent licking and grooming pups over ten days by maternal females, virgin females, and males, the pups in the maternal-virgin and maternal-paternal conditions received more licking and grooming than pups in the maternal only condition (Fig. S3E) (F_(2,99)_ = 17.55, p = 0.0001). Pups were weaned at 3 weeks of age, grouped housed, given standard enrichment (see methods) and at 8 weeks of age experiments were carried out with male and female mice from multiple litters to rule out litter-specific effects.

### Bi-parentally Raised Males Display Enhanced Neurogenesis, which is Passed onto the Next Generation of Male Offspring

**Figure 1:**
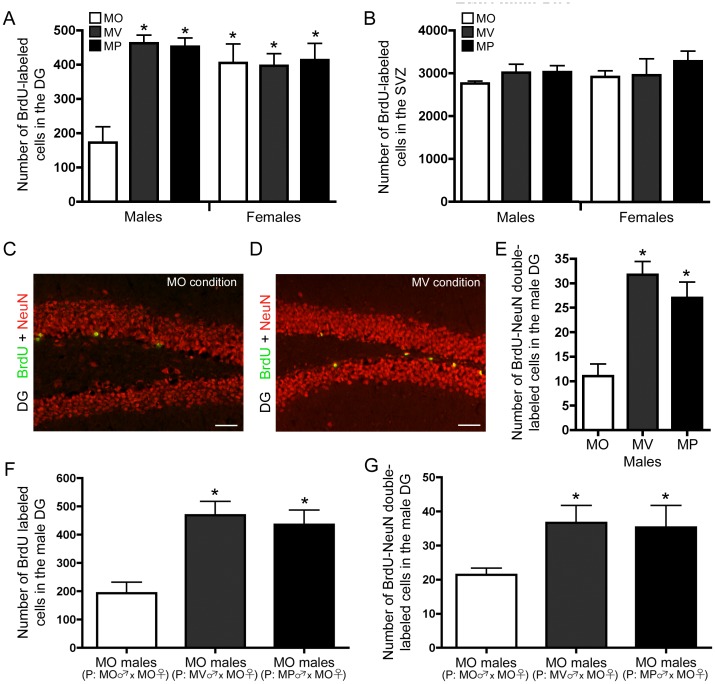
Bi-parentally raised males display enhanced neurogenesis, which is transmitted to the next generation of male offspring. (A) Offspring raised in maternal-only (MO), maternal-virgin (MV) and maternal-paternal (MP) conditions display sexually dimorphic enhancement of adult dentate gyrus (DG) cell proliferation (mean±SEM), where MV and MP males have more BrdU-labeled cells in the DG compared to MO males (n = 5 per group). (B) Cell proliferation in the SVZ is no different among adult male and female offspring raised in different parental conditions (mean±SEM). (C and D) Representative fluorescent micrographs of BrdU-NeuN double labeled cells in the DG of males raised in a single parent environment and bi-parental (MV) environment, respectively. Bars represent 50 µm. (E) MV and MP males show enhanced neurogenesis in the DG (mean±SEM) (n = 5 per group). (F) Males raised in a maternal-only (MO) environment, but sired by MV or MP fathers have more BrdU-labeled cells (mean±SEM) versus MO males sired by MO fathers (n = 5 per group). (G) Males raised in a MO environment, but sired by MV or MP fathers have more BrdU-NeuN double-labeled cells in the DG (mean±SEM) (n = 4 per group).

Male and female mice raised in the three different parental conditions were injected with bromodeoxyuridine (BrdU) to label and quantify the number of proliferating cells within the adult brain. We found a main effect of sex (F_(1,23)_ = 12.79, p = 0.002) regarding the number of BrdU labeled cells in the dentate gyrus. This led us to investigate the effect bi-parental care has on cell proliferation in the adult male brain. We found that adult males raised in the maternal-virgin (MV males) and maternal-paternal (MP males) conditions had approximately 2.5 times more BrdU-labeled cells in the dentate gyrus compared to males raised in the maternal-only condition (MO males) (Fig. 1A) (F_(2,14)_ = 24.88, p<0.0001, posthoc: MO vs MV or MP, p<0.05). This was confirmed by quantifying the number of Ki67-labeled cells in the dentate gyrus (Fig. S4A) (F_(2,9)_ = 52.91, p<0.0001, posthoc: MO vs MV or MP, p<0.05). Further analysis of the dentate gyrus revealed no area differences between MO, MV, and MP males (Fig. S4B). Stereological assessment of the number of BrdU-labeled cells in the dentate gyrus further confirmed that MV and MP males have more BrdU-labeled cells in the dentate gyrus compared to MO males (Fig. S4C) (F_(2.7)_ = 8.649, p = 0.0128, posthoc: MO vs MV or MP, p<0.05). TUNEL analysis revealed no differences in apoptosis in males raised in the three different parental conditions (average number of cells±SEM, n = 6 per group; MO: 6.57±2.44, MV: 14.00±4.48, MP: 10.67±3.31). Surprisingly, cell proliferation in the adult female dentate gyrus was similar regardless of early life parental experience, and also greater than males raised in the maternal-only condition (Fig. 1A) (as previously observed in mice [52]). Moreover, there were no differences in SVZ cell proliferation between MO, MV, and MP males (Fig. 1B).

To determine the differentiated neuronal fate of the newly generated cells in MV and MP males, the number of BrdU-NeuN double-labeled cells in the dentate gyrus was quantified three weeks after BrdU administration. MV and MP males had approximately 2.5 times more BrdU-NeuN double-labeled cells in the dentate gyrus than MO males (Fig. 1C,D,E, Fig. S4D,E) (F_(2,11)_ = 16.84, p = 0.0004, posthoc: MO vs MV or MP, p<0.05). TUNEL analysis revealed no differences in apoptosis in males raised in the three different parental conditions (average number of cells±SEM, n = 5 per group; MO: 3.4±0.87, MV: 2.0±0.71, MP: 2.2±0.73).

We next asked whether increased dentate gyrus neurogenesis in MV and MP males is transmitted to the next generation of offspring. MV and MP males were mated with MO females. Pups were raised in a single parent environment and BrdU was administered at 8 weeks of age. Surprisingly, we found that MO males with MV or MP fathers had approximately 2 times more BrdU-labeled cells (Fig. 1F) (F_(2,11)_ = 10.73, p = 0.0026, posthoc: MO (P: MO female×MO male) vs MO (P: MO female×MV male) or MO (P: MO female×MP male), p<0.05), which was further confirmed using stereological analyses (Fig. S4F) (F_(2,9)_ = 19.94, p = 0.0013, posthoc: MO (P: MO female×MO male) vs MO (P: MO female×MV male) or MO (P: MO female×MP male), p<0.05). Area analysis of the dentate gyrus revealed no difference between MO males with MO, MV, or MP fathers (Fig. S4G). MO males with MV or MP fathers also displayed 1.5 times more BrdU-NeuN double-labeled cells (Fig. 1G) in the dentate gyrus, than MO males with MO fathers (F_(2,10)_ = 6.14, p = 0.0182, posthoc: MO (P: MO female×MO male) vs MO (P: MO female×MV male) or MO (P: MO female×MP male), p<0.05). No differences were observed in the females sired by MO, MV, or MP fathers (average number of cells±SEM, n = 4 per group; MO females (MO female×MO male): 340.00±20.70, MO females (MO female×MV male): 422.75±52.30, MO females (MO female×MP male): 410.25±17.72).

### Bi-parentally Raised Males Display Greater Freezing during Contextual Fear Conditioning

**Figure 2:**
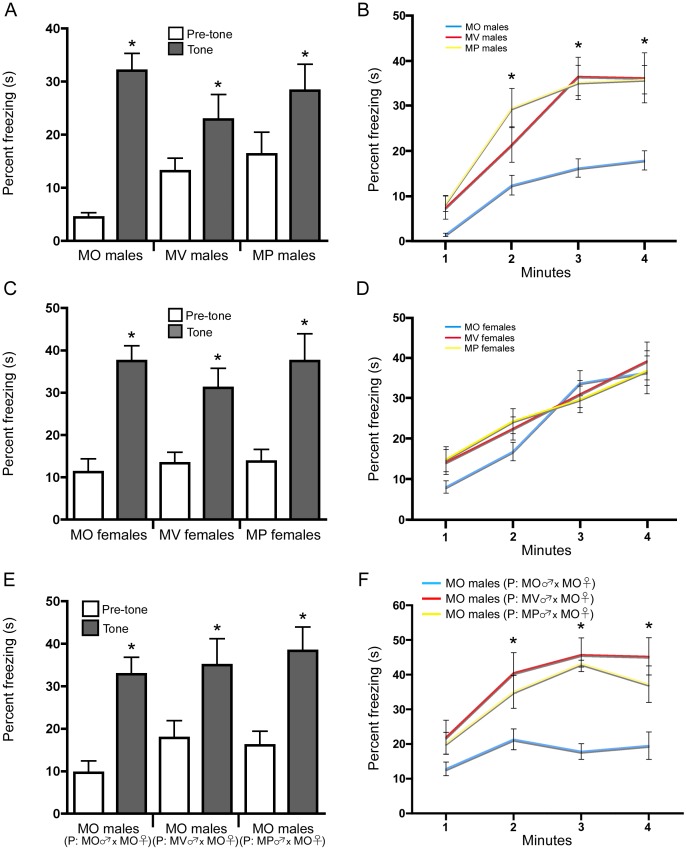
Males with enhanced dentate gyrus neurogenesis display increased freezing during contextual fear conditioning. (A) MO, MV, and MP males show no difference in cue fear conditioning (mean±SEM). (B) MV and MP males display enhanced freezing in contextual fear conditioning, compared to MO males (mean±SEM) (n = 12 per group). (C and D) MO, MV, and MP females, which display no difference in dentate gyrus neurogenesis, show no difference in cue fear conditioning (mean±SEM) or contextual fear conditioning (mean±SEM), respectively. (E) MO males sired by MO, MV, or MP fathers display no difference in cue fear conditioning (mean±SEM). (F) However, MO males sired by MV or MP fathers display a greater percentage of freezing in contextual fear conditioning (mean±SEM) (MO males (P: MO male×MO female) (n = 10), MO males (P: MV male×MO female) (n = 7), and MO males (MP male×MO female) (n = 9)).

Previous studies have shown that newly generated neurons in the dentate gyrus play a role in certain hippocampal-dependent learning and memory tasks [44], [53], [54]. Therefore, we performed various hippocampal learning and memory tasks to assess the behavioural phenotype of MV and MP males, compared to MO males. Adult MO, MV and MP males and females were trained in the cue and contextual fear conditioning protocol outlined in Saxe et al [44]. We found that males and females raised within the three different parental conditions acquired the pairing of a neutral conditioned stimulus (tone) with an aversive unconditioned stimulus (shock) equally (Fig. 2A,C). We found no significant main effect of sex regarding the performance of males and females on cue conditioning. Although there appears to be a difference in the percentage of freezing during the pre-tone phase between MO, MV and MP groups, this is not significant (F_(2,52)_ = 3.676, p = 0.049). We next determined whether the acquisition of this pairing in a distinctive context would differ between MO, MV, and MP males and females. We found a significant main effect of sex (F_(1,52)_ = 4.131, p = 0.037) where further analyses revealed that both MV and MP males displayed significantly more freezing than MO males in the test of context-specific fear (Fig. 2B) (F_(2,52)_ = 7.809, p = 0.01, posthoc: MO vs MV p<0.01 for each of the minutes 3,4; MO vs MP p<0.0001 for each of the minutes 2,3,4), suggesting that enhanced dentate gyrus neurogenesis gives rise to a significant improvement in contextual fear conditioning. These results are similar to those reported for male mice reared in the double-mothering parental environment [15]. Moreover, adult MO, MV and MP females, which do not display differences in dentate gyrus neurogenesis, perform equally in cue and contextual fear conditioning (Fig. 2C,D). Interestingly, when MO males, sired by MV or MP fathers, were tested on cue and contextual fear conditioning, there were no differences observed in cue fear conditioning (Fig. 2E). However, MO males sired by MV or MP fathers displayed a greater degree of contextual fear conditioning (Fig. 2F) (F_(2,69)_ = 2.568, p = 0.0265, posthoc: MO (P: MO female×MO male) vs MO (P: MO female×MV male) p<0.05 for each of the minutes 2,3,4; MO (P: MO female×MO male) vs MO (P: MO female×MP male) p<0.05 for each of the minutes 3,4).

Given the lower percentage of freezing during the pre-tone phase in the cue conditioning task, it may be viewed that MO males have less anxiety than MV and MP males. Therefore, to ensure that increased context-specific freezing behaviour observed in MV and MP males was not due to an increase in anxiety, we examined male mice in the open field, elevated-plus maze and the light-dark choice task [44]. In the open field we found that MV and MP males travel a greater distance than MO males (Fig. S5A) (indicating increased activity and is similar to [55]) (F_(2,30)_ = 7.121, p = 0.0029, posthoc: MO vs MV p<0.001; MO vs MP p<0.05) and display a greater degree of thigmotaxis, as MO males ventured into the core of the arena more than MV and MP males (Fig. S5B) (F_(2,60)_ = 7.997, p = 0.0001, posthoc: MO vs MV core and periphery p<0.01; MO vs MP core and periphery p<0.01). However, the calculated anxiety ratio based on time spent in the open versus closed arms of the elevated-plus maze, as well as the time spent in the light or dark chambers in the light-dark choice task, was no different between the three groups (Fig. S5C,D). Therefore, using the above behavioural measures for anxiety, it appears that anxiety does not differ between MO, MV, and MP males, which is similar to the effect enriched tactile stimulation during early development has on anxiety behaviours [56].

Increased activity has been shown to enhance neurogenesis in the dentate gyrus [20]. Given that MV and MP males traveled a greater distance in the open field, we asked whether there were any differences in home cage activity. By using telemetry to monitor home cage activity over a one-week period, we found no differences in locomotor activity between MO, MV, and MP males (Fig. S5E). We next determined whether enhanced neurogenesis in MV and MP males would give rise to spatial memory differences using the Morris water maze and the Y maze (a place recognition task). We found that MO, MV, and MP males displayed equal ability to acquire where the hidden platform was placed in the water maze using extra-maze spatial cues (Fig. S5F), and displayed similar memory for its location in probe trials conducted one day (Fig. S5G) and one week (Fig. S5H) after acquisition. Moreover, in the Y-maze, males displayed an equal preference to explore the novel arm of the maze (Fig. S5I). Therefore, enhanced adult dentate gyrus neurogenesis in MV and MP males gives rise to improved performance on specific hippocampal-dependent behavioural tasks.

### Bi-parentally Raised Females Display Enhanced White Matter Oligodendrocyte Production, which is Passed onto the Next Generation of Female Offspring

**Figure 3:**
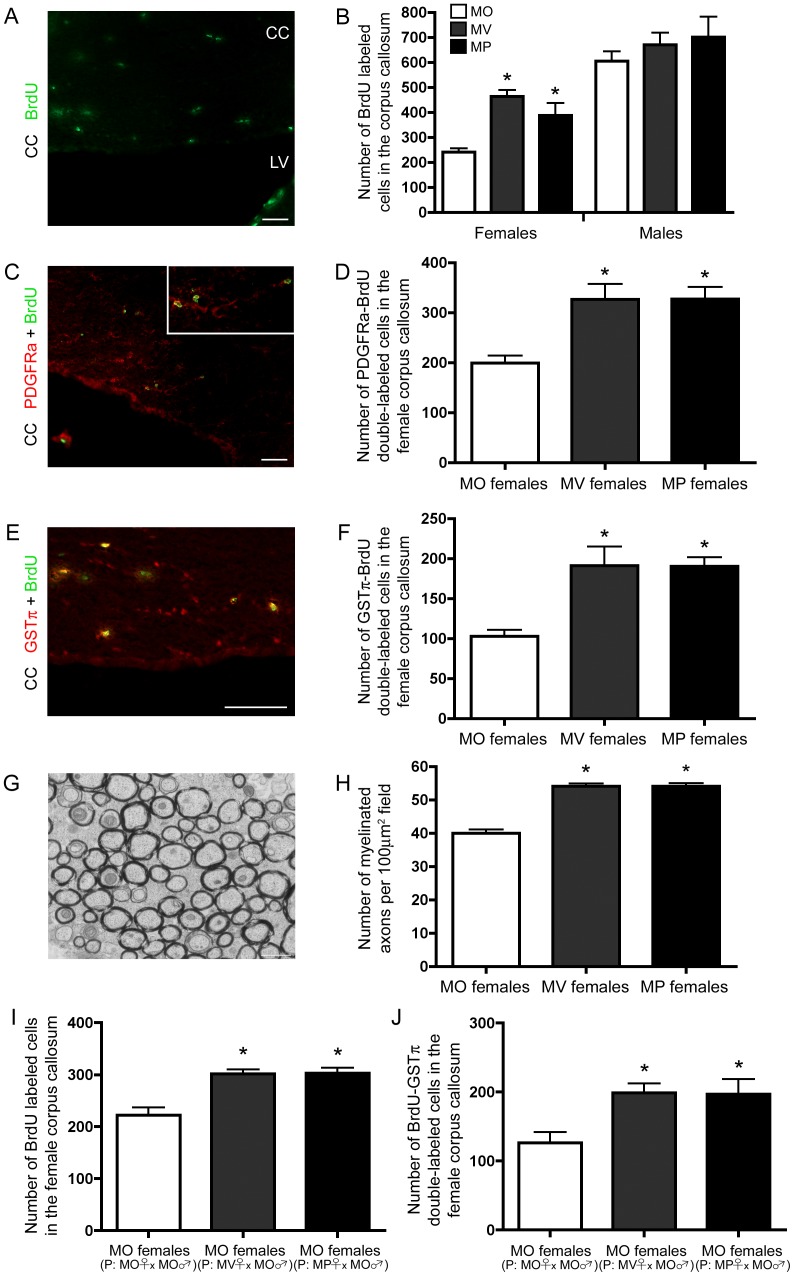
Females raised in a bi-parental environment have more newly generated myelinating oligodendrocytes and myelinated axons in the corpus callosum, which is transmitted to the next generation of female offspring. (A) Offspring raised in maternal-only (MO), maternal-virgin (MV) and maternal-paternal (MP) conditions, display sexually dimorphic enhancement of adult corpus callosum (CC) cell proliferation. Fluorescent micrograph of BrdU-labeled cells in the CC. Bar represents 50 µm. (B) MV and MP females have increased numbers of BrdU-labeled cells in the CC compared to MO females (mean±SEM) (n = 4 per group). (C) Fluorescent micrograph of PDGFRa-BrdU double-labeled cells in the CC. Bar represents 50 µm. (D) MV and MP females display more PDGFRa-BrdU double-labeled cells in the CC than MO females (mean±SEM) (n = 4 per group). (E) Fluorescent micrograph of GSTπ-BrdU double-labeled cells in the CC. Bar represents 50 µm. (F) MV and MP females display enhanced oligodendrogliogenesis in the CC compared to MO females (mean±SEM) (n = 4 per group). (G) Electron micrograph of axons in the genu of CC. (H) There are more myelinated axons in the genu of CC in MV and MP females than MO females (mean±SEM) (n = 42 (MO images), n = 56 (MV images) and n = 56 (MP images)). (I) Females raised in a MO environment, but sired by MV or MP mothers have more BrdU-labeled cells (mean±SEM) (MO females (n = 4 per group). (J) Females raised in a MO environment, but sired by MV or MP mothers have more BrdU-GSTπ double-labeled cells in the corpus callosum (mean±SEM) (n = 4 per group).

Given that bi-parental care can modulate adult neurogenesis, we next asked whether the generation of new myelinating oligodendrocytes, is also modulated by bi-parental care. We found a main effect of sex (F_(1,15)_ = 66.794, p = 0.00001), which led us to further investigate the effect bi-parental care has on the female brain. We found that MV and MP females had approximately 2 times more BrdU-labeled cells in the corpus callosum than MO females (Fig. 3A,B) (F_(2,9)_ = 11.90, p = 0.0030, posthoc: MO vs MV or MP, p<0.05). Surprisingly, cell proliferation in the male corpus callosum was similar regardless of early life parental care experience and approximately 2.5 times greater than MO females (Fig. 3B). Moreover, there were no differences in the number of TUNEL-expressing cells in the corpus callosum between MO, MV, and MP females (average number of cells±SEM, n = 6; MO: 13.5±3.96, MV: 18.33±1.71, MP: 11.00±2.11). To investigate whether increased cell proliferation persists in another central nervous system white matter region of MV and MP females, we quantified the number of BrdU-labeled cells in the spinal cord. MV and MP females had approximately 1.5 times more BrdU-labeled cells in the spinal cord than MO females (Fig. S6A,B) (F_(2,15)_ = 7.389, p = 0.0058, posthoc: MO vs MV or MP, p<0.05).

To determine the differentiated phenotype of newly generated cells in the female corpus callosum, the number of BrdU-platelet derived growth factor receptor alpha (PDGFRa) double-labeled cells were quantified, where PDGFRa is a marker for immature oligodendrocytes. There were approximately 1.5 times more BrdU-PDGFRa double-labeled cells in the corpus callosum of MV and MP females, compared to MO females (Fig. 3C,D) (F_(2,9)_ = 9.260, p = 0.0065, posthoc: MO vs MV or MP, p<0.05). To determine whether MV and MP females had increased number of mature oligodendrocytes in the corpus callosum, we counted the number of BrdU-glutathione S-transferase-π (GSTπ) double-labeled cells 12 days after administering BrdU. We found that both MV and MP females had 2 times more BrdU-GSTπ double-labeled cells in the corpus callosum than MO females (Fig. 3E,F) (F_(2,9)_ = 10.98, p = 0.0039, posthoc: MO vs MV or MP, p<0.05). Given that newly generated oligodendrocytes have the potential to contribute to myelination, we asked whether more myelinated axons were present in the genu of the corpus callosum of MV and MP females, compared to MO females. We first determined whether the area of the genu of corpus callosum was different between MO, MV, and MP females by staining white matter in the brain with eriochrome cyanine. Using ImageJ to quantify the area of interest, we found no gross anatomical white matter differences between MO, MV, and MP females (Fig. S6C,D). Using electron microscopy we found that the number of myelinated axons was 1.4 times greater in MV and MP females than MO females (Fig. 3G,H) (F_(2,151)_ = 67.39, p = 0.0001, posthoc: MO vs MV or MP, p<0.05). Therefore, bi-parental care increases the number of new myelinating oligodendrocytes and myelinated axons in the adult female corpus callosum.

Given that enhance dentate gyrus neurogenesis in males is transmitted to the next generation of male offspring, we asked whether increased numbers of new myelinating oligodendrocytes in the corpus callosum of MV and MP females is transmitted to the next generation of female offspring. Females sired by MV or MP mothers had approximately 1.5 times more BrdU-labeled cells (Fig. 3I) (F_(2,13)_ = 16.05, p = 0.0003, posthoc: MO (P: MO female×MO male) vs MO (P: MV female×MO male) or MO (P: MP female×MO male), p<0.05) and BrdU-GSTπ double-labeled cells (Fig. 3J) (F_(2,13)_ = 6.187, p = 0.0178, posthoc: MO (P: MO female×MO male) vs MO (P: MV female×MO male) or MO (P: MP female×MO male), p<0.05) in the corpus callosum than MO females sired by MO mothers. No differences were observed in males sired by MO, MV, or MP mothers (average number of cells±SEM, n = 5; MO males (MO female×MO male): 520.00±33.87, MO males (MV female×MO male): 621.20±36.48, MO males (MP female×MO male): 524.20±30.27).

### Bi-parentally Raised Females Display Greater Bilateral Motor Coordination and Preference for Social Investigation

**Figure 4:**
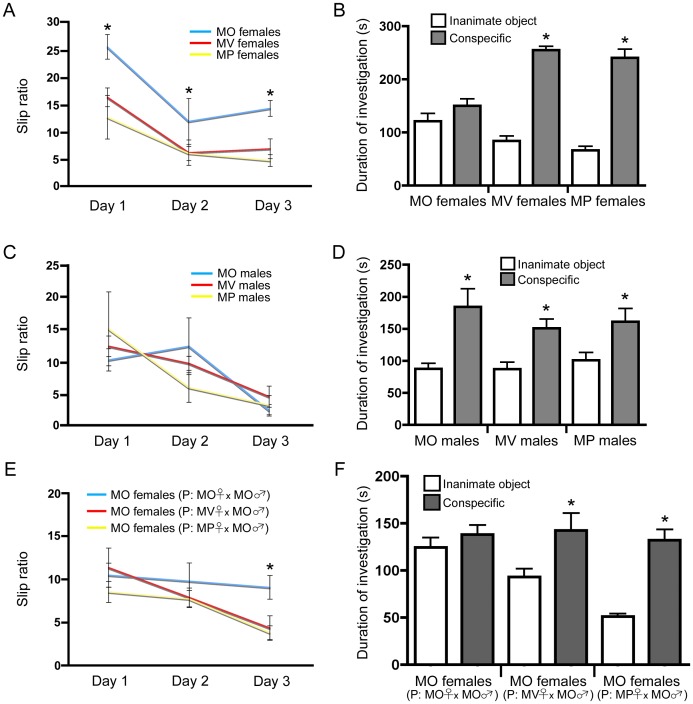
Females with enhanced numbers of newly generated myelinating oligodendrocytes and myelinated axons in the corpus callosum display greater bilateral motor coordination and preference for social investigation. (A) MV and MP females display less slipping on the horizontal ladder rung task than MO females (n = 11 per group). (B) MV and MP females also show a greater preference for social investigation as they spend more time investigating a novel conspecific than an inanimate object (mean±SEM) (n = 10 per group). (C and D) MO, MV, and MP males, which do not display enhanced oligodendrogliogenesis, show no difference in performance on the horizontal ladder rung. MO, MV, and MP males all demonstrate a greater preference for investigating the novel conspecific versus the inanimate object (mean±SEM) (n = 8 per group). (E) MO females sired by MV or MP mothers demonstrate less slipping on the horizontal ladder rung over time (mean±SEM) (n = 10 per group). (F) MO females sired by MV or MP mothers display greater investigation towards a novel conspecific (mean±SEM) (MO females (P: MO female×MO male) (n = 10), MO females (MV female×MO male) (n = 11) and MO females (MP female×MO male) (n = 13)).

The corpus callosum is thought to be involved in establishing cerebral laterality and mediating interhemispheric crosstalk and spatial coupling between the limbs [57]. Further, individuals with autism spectrum disorders exhibit white matter deficiencies and thus, the corpus callosum is also thought, in part, to be involved in social behaviours [57], [58]. Interestingly, enriched environments increase myelination of the corpus callosum [59], whereas social deprivation leads to less complex oligodendrocyte morphology and reduced myelination in the prefrontal cortex [60]. Therefore, we asked whether a greater number of oligodendrocytes and myelinated axons in the corpus callosum would give rise to differences in bilateral coordination and social behaviour. Adult female mice were trained to walk along the horizontal ladder rung with evenly spaced rungs and subsequently tested for their ability to walk along unevenly spaced rungs. A slip ratio was calculated based on performance. We determined that MV and MP females were more proficient at walking along the unevenly spaced rungs than MO females (Fig. 4A) (F_(2,22)_ = 33.206, p = 0.01, posthoc: MO vs MV p<0.01 on days 1,2,3; MO vs MP p<0.01 on days 1,2,3). Interestingly, there was also a main effect of sex on ladder rung performance (F_(1,42)_ = 21.562, p = 0.00034), where females outperformed males. The preference for social investigation of females was then examined based on their duration of investigating an inanimate object versus a same-sex, age-matched, novel conspecific (presented simultaneously). MV and MP females showed a greater degree of interest in the novel conspecific than the MO females (Fig. 4B) (F_(2,23)_ = 17.831, p = 0.0021), whereas MO females showed a greater degree of investigation for the inanimate object (F_(2,23)_ = 7.505, p = 0.003). MO, MV, and MP males tested for bilateral coordination and social preference displayed equal performance on both tasks (Fig. 4C,D), which would be expected as no differences in the number of newly generated myelinated oligodendrocytes were observed in males. Moreover, MO females sired by MV or MP mothers also displayed greater bilateral coordination (Fig. 4E) (F_(2,29)_ = 2.756, p = 0.0370, posthoc: MO (P: MO female×MO male) vs MO (P: MV female×MO male) on day 3 p<0.01; MO (P: MO female×MO male) vs MO (P: MP female×MO male) on day 3 p<0.01). MO females sired by MO mothers displayed a greater degree of investigation for the inanimate object (F_(2,31)_ = 24.947, p = 0.00001) (Fig. 4F). MO females sired by MO, MV or MP mothers display an equal degree of investigation for the conspecific. However, MO females sired by MV and MP mothers show more investigation towards the conspecific than the inanimate object compared to MO females sired by MO mothers (posthoc: MO (P: MV female×MO male) and MO (P:MP female×MO male) females inanimate vs conspecific p<0.05).

To rule out any differences in general anxiety between MO, MV, and MP females (which may affect performance on the ladder rung and sociability tasks), behaviour in the open field was observed. We found that females display equal levels of thigmotaxic behaviour (Fig. S7A). Interhemispheric crosstalk through the corpus callosum may be important for tasks involving higher cognitive functioning [57], [58]. Therefore, we sought to determine whether differences in the Morris water maze, passive-avoidance, and pre-pulse inhibition tasks would be observed between MO, MV, and MP females. We found that there was no difference in acquiring where the hidden platform was placed in the water maze using extra-maze spatial cues (Fig. S7B), and all females displayed similar memory for where the hidden platform was during the probe trial (Fig. S7C). Similarly, MO, MV, and MP females performed equally on the passive-avoidance task, (which assesses short- and long- term memory) (Fig. S7D). Further, females displayed no differences in sensorimotor gating when using the prepulse inhibition task (Fig. S7E). Therefore, MO, MV, and MP females display similar levels of anxiety and sensorimotor gating, and performed equally on various learning and memory tasks.

## Discussion

Early life experiences can alter brain and behavioural development that may result in life-long functional alterations [1], [2], [5], [16]. Adverse experiences have been demonstrated to increase the propensity of psychosocial and neuropsychiatric diseases [61], whereas enriched experiences may increase resilience to stressful psychological and biological events [62]. Interestingly, behavioural phenotypes and associated plasticity changes arising from different early life experiences can be sexually dimorphic and transmissible across generations [1], [2], [5], [14], [16], [17], [18]. Our study shows that bi-parental care enhances adult neural cell genesis in a sexually dimorphic manner, which gives rise to specific behavioural phenotypes. Surprisingly, the enhanced neural cell genesis and associated changes in behaviour identified are transmitted to the next generation of offspring.

Previous studies have shown that adult male dentate gyrus neurogenesis is reduced as a result of maternal separation [23]. However, if males are reared in a communal nest [24], by a mother displaying more maternal care [25] or in an enriched postnatal environment [26], the survival of newly generated dentate gyrus neurons is enhanced. Moreover, an enriched postnatal environment has also been shown to increase the maturation of new dentate gyrus neurons prior to weaning [63]. Given that prior studies were restricted to males, the impact differential levels of parental care and enriched postnatal environments have on adult female dentate gyrus neurogenesis remains largely unexplored. In our study, the levels of adult female dentate gyrus neurogenesis were similar to those of bi-parentally raised males (regardless of the female’s parenting environment), suggesting that reduced parental care specifically affects male neurogenesis. When we compared the parental care behaviours in the different environments, we showed that pups raised in a bi-parental environment experienced more licking and grooming than pups raised in a maternal only environment. These results are similar to the results reported in D’Amato, which demonstrated that double-mothering increases the amount of licking and grooming experienced by the pups versus pups reared by dams alone [15]. It has been demonstrated in rats that lactating females preferentially lick and groom male pups over females [64]. As such, it is plausible that the fathers or virgin foster moms present in the MV and MP rearing environments may also preferentially lick male offspring, thus potentially contributing to the sex differences displayed in dentate gyrus neurogenesis (although preferential licking of males from alloparental and paternal conspecifics warrants further investigation). Therefore, licking and grooming may induce a physiological response to trigger enhanced neurogenesis in the male dentate gyrus. Moreover, the selective enhancement in contextual fear memory of bi-parentally raised adult males, further substantiates a role for adult dentate gyrus neurogenesis in hippocampal-dependent processes associated with emotional responses [65].

We acknowledge that the specific function of adult born neurons in the dentate gyrus remains controversial. The attempt to address this controversy is beyond the scope of this study. Previous research using focal X irradiation and genetic ablation of GFAP positive dividing neural progenitors in the mouse hippocampus revealed an impairment in contextual fear conditioning, but sparing of spatial memory [44]. Moreover, further irradiation studies that tease apart the dependency of newly generated neurons in the dentate gyrus on context, have not only confirmed the role of dentate gyrus neurogenesis in contextual fear conditioning, but also points to its importance for learning temporally displaced events within a specific task and emotionally relevant contextual information [66], [67]. Recently, it has also been demonstrated that when ERK5 is conditionally deleted, dentate gyrus neurogenesis is disrupted [68] and subsequently leads to impairment in the initial establishment and maintenance of contextual fear memory [69]. Therefore, newly generated neurons of the dentate gyrus may function in context-dependent learning processes which incorporates high emotional arousal.

Additionally, it is not unreasonable to consider that newly generated dentate gyrus neurons are involved in a broad range of hippocampal function. Indeed, an enriched environment increases dentate gyrus neurogenesis and has been shown to improve spatial learning performance on the Morris water maze, when compared to animals raised in an isolated environment [70]. Although, we do not see differences in spatial learning between bi-parentally raised and maternal only raised mice, this may be attributed to the overall impact of housing conditions. Regardless of parental upbringing, the mice in our study were all socially housed with home cage enrichment, whereas an isolated environment provided no social or home cage enrichment in Nilsson et al [70]. In line with the impact of enriched environments on dentate gyrus neurogenesis, voluntary running in socially housed rodents has been shown to increases dentate gyrus neurogenesis [71] and leads to an improvement in spatial learning [72]. Interestingly, mice bred to be highly motivated for increased voluntary running displayed enhanced neurogenesis above control mice with access to running wheels [73]. However, the enhanced neurogenesis in these selectively bred mice did not improve their spatial learning performance beyond what was displayed by control mice with access to running wheels [73]. Thus, suggesting a ceiling effect for the positive correlation of enhanced dentate gyrus neurogenesis and improved spatial learning. Further, a study that pharmacologically blocked dentate gyrus neurogenesis or angiogenesis during voluntary running, demonstrated that inhibiting angiogenesis impairs spatial learning and memory performance, whereas inhibiting neurogenesis resulted in the opposite [74]. Despite these conflicting results, it is possible that the dependency of newly generated dentate gyrus neurons on spatial learning and memory can be teased out using more complex spatial learning and memory tasks. Indeed, Dupret et al demonstrated that double transgenic mice, where neural progenitors of the dentate gyrus were selectively ablated, displayed no performance deficit in contextual fear conditioning and spatial learning when spatial training was acquired by consistent release points (starting points) at the beginning of every trial using the Morris water maze task [75]. However, when release points where varied across each trial, the double transgenic mice displayed impaired spatial learning [75]. As previously reported in the irradiation and enriched environment studies (as well as this study) where the Morris water maze was employed to assess the function of newly generated neurons in the dentate gyrus, the location of the release points were varied every trial during training [44], [70], [72], [73].

In line with using more complex spatial learning procedures to assess the dependency of newly generated neurons on spatial learning, a study by Garthe et al used a reversal protocol of the water maze task on mice with temozolomide (TMZ) ablated dentate gyrus neurogenesis [76]. Both TMZ-treated and control mice successfully learned to locate the hidden platform during the first three days of training. On the fourth day when the platform was moved to the opposite quadrant, both groups spent significantly more time in the quadrant where the platform was previously located. However, when mice were assessed two days after reversal training, the TMZ-treated mice failed to learn where the new platform was located. Given that the average number of BrdU labeled cells that we quantified in the dentate gyrus of MO, MV and MP males are similar to the numbers reported in TMZ treated mice, and that we only see a ∼2.5 times reduction in BrdU labeled cells in the MO males compared to ∼5 times reduction reported for the TMZ-treated mice, it is possible that we will see no difference in reversal learning between MV and MP males versus MO males. The possible differences in BrdU labeled cells may be attributed to the different BrdU-labeled protocols used. However, our BrdU protocol has given consistent BrdU counts in control animals across a number of studies published from our lab (see [50], [77], [78]). The average number of BrdU labeled cells in the dentate gyrus reported for control mice in Garthe et al are greater than the average number of BrdU labeled cells in the dentate gyrus reported for mice that have access to voluntary physical exercise and environmental enrichment [79], as well as mice selectively bred to display increased voluntary running behaviour [73]. The BrdU cell counts reported in our study are more in line with the mice not selectively bred to display increased voluntary running behaviour (controls). Therefore, the housing and enrichment conditions used by Garthe et al may be very different than those employed in our study. Moreover, the housing and enrichment used in Garthe et al may have also contributed to the findings observed in their study. However, despite the apparent differences in baseline neurogenesis in the dentate gyrus between our study and Garthe et al, it is possible that a more qualitative assessment of strategies used in the Morris water maze task will identify aspects of spatial learning that are different between MO, MV and MP males. This warrants further investigation in the future.

As such, newly generated neurons of the dentate gyrus may be involved in a number of different hippocampal-dependent processes that involve spatial, emotional and temporal aspects. Indeed Stone et al found that both adult generated and developmentally generated dentate granule cells were equally likely to contribute to hippocampal-dependent processing in the Morris water maze task (using non-reversal protocol) and contextual fear conditioning [80]. Further, it may be possible that the positional integration of newly generate neurons may preferentially influence the role specific neurons play in modulating and/or contributing to the various functional aspects the hippocampus is involved in [65], [81]. A separate study will be required to address the relationship between hippocampal plasticity in the form of newly generated neurons, and the multifaceted nature of hippocampal-dependent processing.

Sexual dimorphism in the adult brain white matter has been the subject of intense investigation and may explain differential gender susceptibility to certain myelin diseases [82]. Although previous studies have pointed to an enriched environment contributing to enhanced corpus callosum myelination [59] and a critical period of social experience necessary for prefrontal cortical myelination [60], the influence bi-parental care has on differential oligodendroctye production in adult males and females are unknown. Our findings demonstrate that bi-parental care enhances adult female oligodendrocyte production to levels similar to adult males, whose white matter cell genesis appears to be unchanged by bi-parental care. The increased licking and grooming experienced by females raised in a bi-parental environment may trigger a physiological response to contribute to enhanced white matter cell genesis. This suggests that reduced parental care may, in part, contribute to fewer myelinated axons and a smaller corpus callosum in females compared to males. Our findings of enhanced bilateral motor coordination and preference for social investigation in adult females raised in a bi-parental environment supports the hypothesis of human white matter involvement in motor performance [83], a characteristic pathology associated with autism [58], and the impact bi-manual childhood activities have on white matter development [84]. However, correlations between sexual dimorphism, oligodendrocyte production and behaviour, require future investigation.

We have demonstrated that pups raised in a bi-parental environment experience more licking and grooming. However, the brain-specific physiological response induced by this particular aspect of parental care is unknown and whether such physiological response is different in males and females also warrants further investigation. Future studies will examine the mechanisms associated with gender-specific transmissibility of enhanced neural cell genesis and the associated behavioural phenotype to the next generation. Given the increasing evidence for the transmissibility of socially driven phenotypic variations across generations [2], [16], [17], our study provides the first glimpse into the neurobiological link between re-occurring intergenerational behavioural outputs and brain plasticity. It is possible that bi-parental care increases the expression of specific neurotrophic factors or alters reproductive hormonal levels to influence neural stem cell proliferation and cell fate decisions in the germinal regions of the adult mammalian brain [1], [2], [5], [16]. Moreover, just as changes in maternal behaviour has been associated with epigenetic programming [85], perhaps bi-parental care also gives rise to post-translational modifications on specific proteins and/or DNA methylation of specific genes to regulate the adult neural stem cell lineage. Thus, epigenetic changes may, in part, account for the generational transmission of enhanced neural cell genesis and associated behavioural phenotypes, where the bi-parental paradigm employed in our study can serve as a viable model to identify how neural precursors adopt persistent epigenetic modifications. Elucidating such mechanisms will undoubtedly contribute to our understanding of how early life experience can induce persistent and transmissible changes in adult brain plasticity and behaviour.

## Supporting Information

Figure S1 **Paradigm of different early life parental care conditions.** Female mice are mated with male mice, where mating is detected by the presence of a plug. Pregnant females are then separated into different conditions: pregnant females are placed into an individual cage to raised pups alone (Maternal only condition); pregnant females are placed in individual cages with an age-matched virgin female for the duration of pregnancy and when pups are born, virgin females act as “foster-parents” to the pups (Maternal-virgin condition); individual pairs of males and females are mated and remain together for the duration of pregnancy and after the pups are born (Maternal-paternal condition). Pups are left in the three different conditions until the age of weaning (21 days), upon which they are separated into individual same-sex cages. Parental observations were conducted from P0-P10 four times a day for 15 minutes each observation. Subsequent histological and behavioural experiments are conducted at 8 weeks of age onwards using mice from multiple litters to rule out litter-specific effects.
(TIF)

Figure S2 **Observation of parental behaviours exhibited by maternal females in the different parental conditions.** Maternal females within the maternal only (MO), maternal-virgin (MV), and maternal-paternal (MP) conditions did not differ in the amount of time spent conducting specific parental behaviours of: (A) arch back nursing, (B) licking and grooming, (C) nest building, (D) resting in nest, and (E) off nest, from postnatal day (P) 0– P10.
(TIF)

Figure S3 **Observation of parental behaviours exhibited by virgin females and males, and the total amount of licking and grooming directed towards developing pups in the different parental conditions.** Virgin females and males in the maternal-virgin and maternal-paternal conditions, respectively did not differ in the amount of time spent conducting specific parental behaviours of: (A) licking and grooming, (B) nest building, (C) resting in nest, and (D) off nest, from postnatal day (P) 0– P10. (E) The average licking and grooming experienced by pups over ten days is significantly greater in the maternal-virgin (MV) and maternal-paternal (MP) conditions versus the maternal-only (MO) condition.
(TIF)

Figure S4 **Enhanced parental care increases cell proliferation in the adult male dentate gyrus (DG).** (A) Adult males raised in the maternal-virgin (MV) and maternal-paternal (MP) environments, have greater numbers of Ki67-labeled cells (mean±SEM) in the DG than adult males raised in a maternal-only (MO) environment. (B) The area (µm^2^) of the DG does not differ between MO, MV and MP males (mean±SEM). (C) Stereological quantification of BrdU-labeled cells in the DG revealed that MV and MP males have a greater number of BrdU-labeled cells than MO males (mean±SEM). (D) Representative fluorescent micrograph of BrdU-NeuN double-labeled cells in the DG of males raised in the maternal paternal environment. (E) Representative BrdU-NeuN double-labeled cells in the DG at higher magnification (40X). Bars in both D and E represent 50 µm. (F) Stereological analyses demonstrated that the number of BrdU-labeled cells in the DG is greater in MO males that are offspring of MV and MP fathers, compared to MO males that are offspring of MO fathers (mean±SEM). (G) The area (µm^2^) of the DG does not differ between MO males that are offspring of MO, MV, or MP fathers (mean±SEM).
(TIF)

Figure S5 **Assessment of general anxiety and spatial memory of adult males.** (A) Males raised in maternal-virgin (MV) (n = 11) and maternal-paternal (MP) (n = 10) environments travel a greater distance in the open field compared to males raised in a maternal-only (MO) environment (n = 12) (mean±SEM). (B) MO males spend a greater percentage of time in the core of the arena compared to MV and MP males. However, MO, MV, and MP males, all spend a greater amount of time in the periphery of the open field (mean±SEM). (C) The general anxiety ratio of MO (n = 6), MV (n = 7), and MP (n = 7) males did not differ when measured using the elevated plus maze (mean±SEM). (D) When MO (n = 6), MV (n = 7), and MP (n = 7) males were placed in the light-dark choice task, they exhibited equal preference to spend more time in the dark-side of the chamber (mean±SEM). (E) MO, MV, and MP males show no difference in home cage activity (mean±SEM) (n = 6 for each group). (F) All males display equal ability to find a hidden platform in the Morris water maze during training (mean±SEM) (n = 8 for each group). (G and H) All males display equal time spent in the target quadrant where the hidden platform was previously placed when assessed one day after training, as well as seven days after training, respectively (mean±SEM). (I) The Y-maze was used to assess place recognition in MO (n = 6), MV (n = 7), and MP males (n = 7), which all showed equal levels of entry into the novel arm of the maze (mean±SEM).
(TIF)

Figure S6 **Enhanced parental care increases cell proliferation in the adult female spinal cord. Size of the adult corpus callosum is not affected in females raised in different parental care environments.** (A) Adult females raised in maternal-virgin (MV) and maternal-paternal (MP) environments, have greater numbers of BrdU-labeled cells in the spinal cord (mean±SEM) than adult females raised in a maternal-only (MO) environment. (B) Fluorescent micrograph of BrdU-labeled cells in the spinal cord. Bar represents 50 µm. (C) The area of the adult genu of corpus callosum of MO, MV and MP females does not differ (mean±SEM) (n = 6 for each group). (D) Region of analysis depicted on a sagittal section stained with Eriochrome cyanine.
(TIF)

Figure S7 **Assessment of general anxiety, spatial memory, and prefrontal cortex-dependent learning and memory in adult females.** (A) In the open field, females raised in maternal-only (MO) (n = 8), maternal-virgin (MV) (n = 8), and maternal-paternal (MP) (n = 10) environments spent more time in the periphery than the core or middle of the arena (mean±SEM). (B) MO (n = 8), MV (n = 8), and MP (n = 10) females displayed equal ability to locate a hidden platform in the Morris water maze. (C) A probe trial conducted one day after training in the Morris water maze revealed no difference in the percentage of time MO, MV, and MP females spent in the quadrant where the hidden platform was previously located (mean±SEM). (D) MO (n = 8), MV (n = 8), and MP (n = 10) females equally demonstrate a memory to not cross to the novel side of the chamber in the passive-avoidance task the day after training (Day 2) and one week after training (Day 7) (mean±SEM). (E) MO (n = 8), MV (n = 8), and MP (n = 10) females show no difference in percent pre-pulse inhibition (mean±SEM).
(TIF)
