# Supplementary figures and images for: Bi-Parental Care Contributes to Sexually Dimorphic Neural Cell Genesis in the Adult Mammalian Brain
Source: PLoS One. 2013 May 1;8(5):e62701. doi: 10.1371/journal.pone.0062701 (PMC3641101; doi:10.1371/journal.pone.0062701)

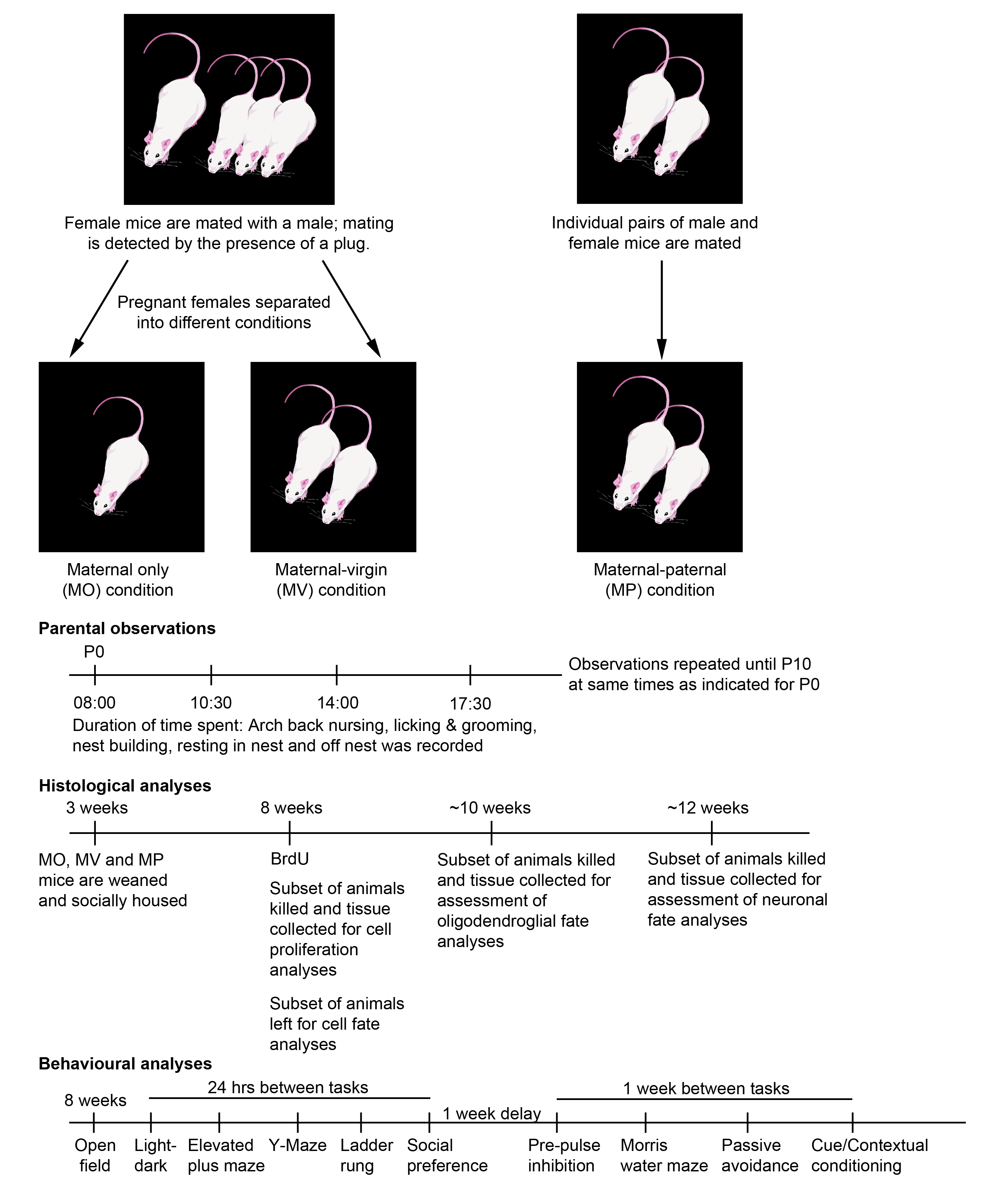

Supplement: Figure S1 — Paradigm of different early life parental care conditions. Female mice are mated with male mice, where mating is detected by the presence of a plug. Pregnant females are then separated into different conditions: pregnant females are placed into an individual cage to raised pups alone (Maternal only condition); pregnant females are placed in individual cages with an age-matched virgin female for the duration of pregnancy and when pups are born, virgin females act as “foster-parents” to the pups (Maternal-virgin condition); individual pairs of males and females are mated and remain together for the duration of pregnancy and after the pups are born (Maternal-paternal condition). Pups are left in the three different conditions until the age of weaning (21 days), upon which they are separated into individual same-sex cages. Parental observations were conducted from P0-P10 four times a day for 15 minutes each observation. Subsequent histological and behavioural experiments are conducted at 8 weeks of age onwards using mice from multiple litters to rule out litter-specific effects. (TIF) [file pone.0062701.s001.tif]

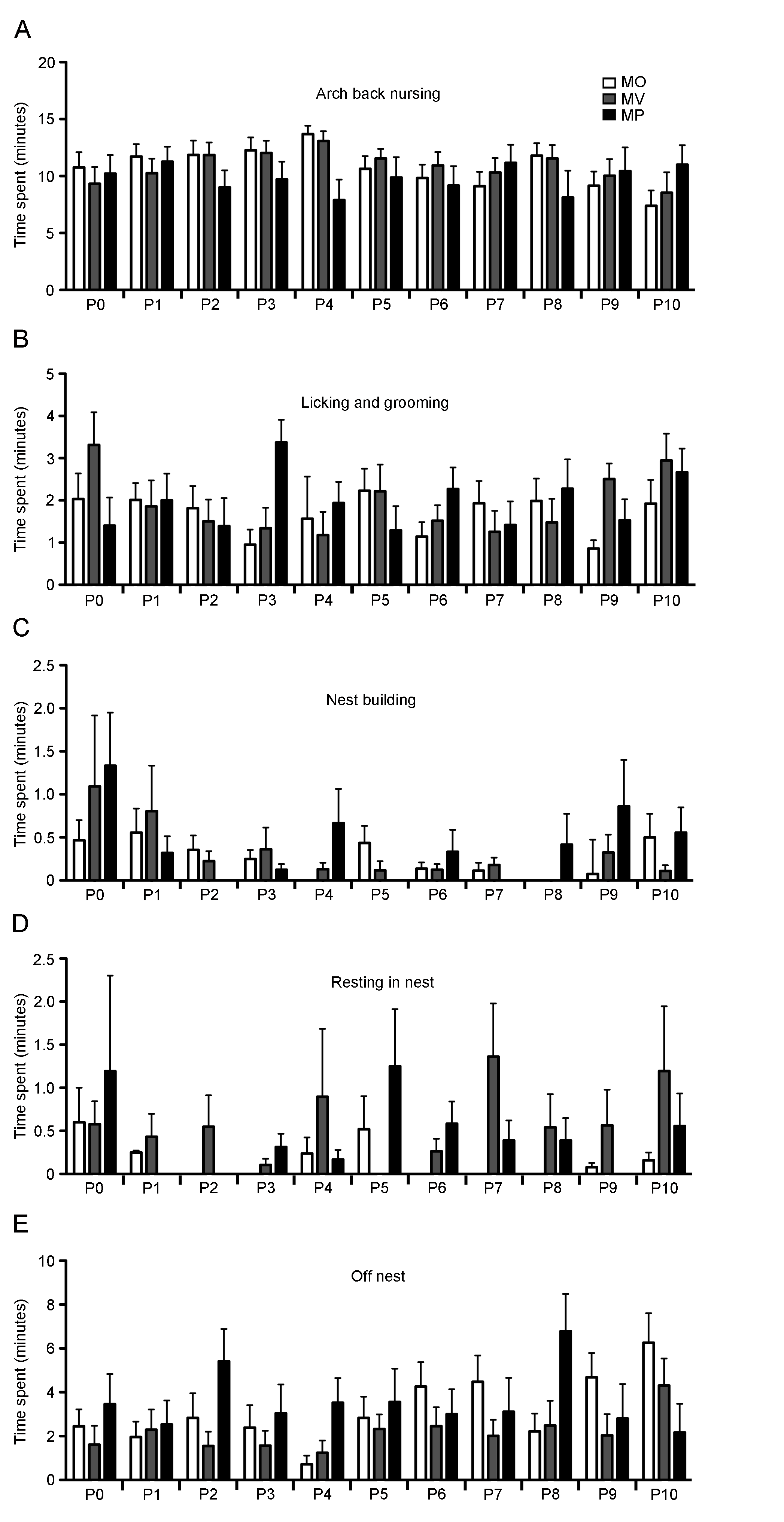

Supplement: Figure S2 — Observation of parental behaviours exhibited by maternal females in the different parental conditions. Maternal females within the maternal only (MO), maternal-virgin (MV), and maternal-paternal (MP) conditions did not differ in the amount of time spent conducting specific parental behaviours of: (A) arch back nursing, (B) licking and grooming, (C) nest building, (D) resting in nest, and (E) off nest, from postnatal day (P) 0– P10. (TIF) [file pone.0062701.s002.tif]

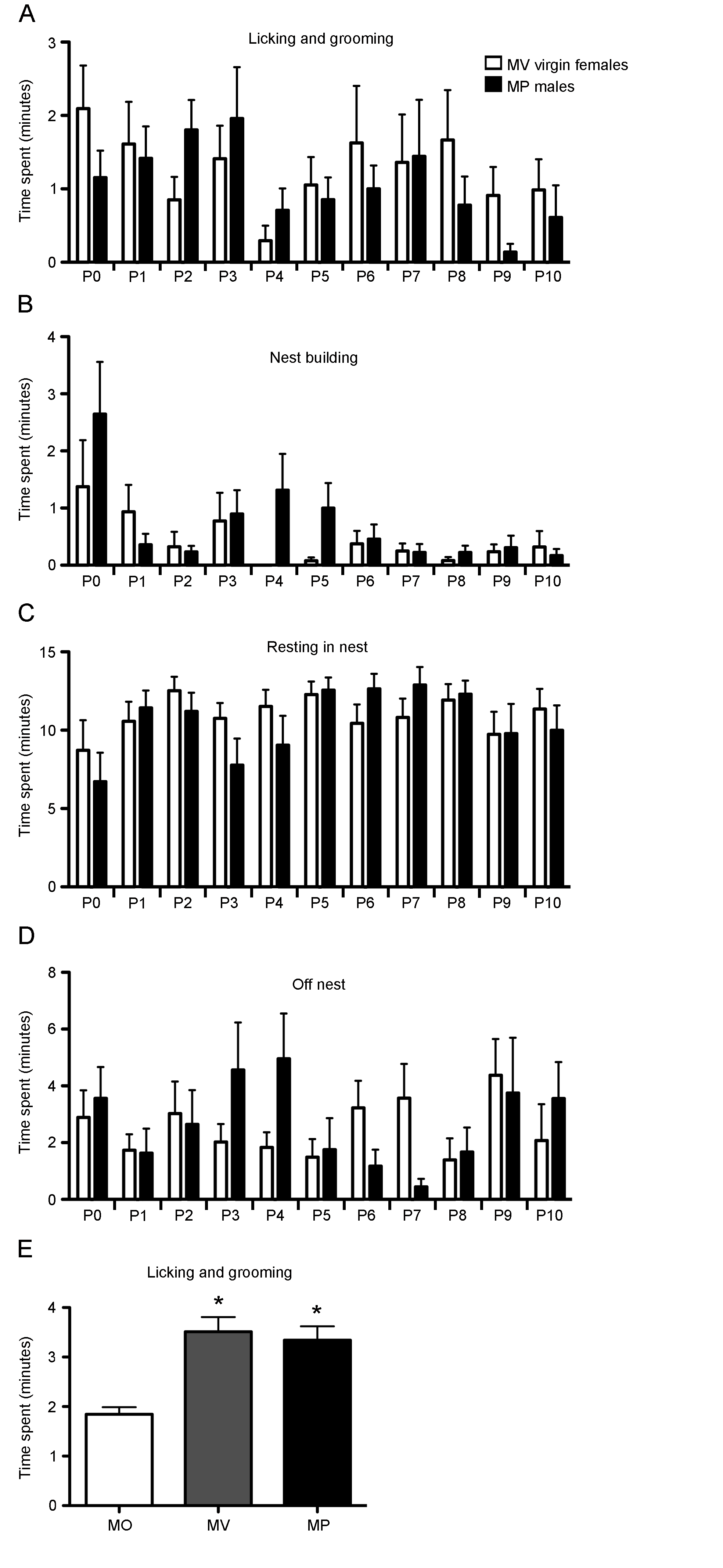

Supplement: Figure S3 — Observation of parental behaviours exhibited by virgin females and males, and the total amount of licking and grooming directed towards developing pups in the different parental conditions. Virgin females and males in the maternal-virgin and maternal-paternal conditions, respectively did not differ in the amount of time spent conducting specific parental behaviours of: (A) licking and grooming, (B) nest building, (C) resting in nest, and (D) off nest, from postnatal day (P) 0– P10. (E) The average licking and grooming experienced by pups over ten days is significantly greater in the maternal-virgin (MV) and maternal-paternal (MP) conditions versus the maternal-only (MO) condition. (TIF) [file pone.0062701.s003.tif]

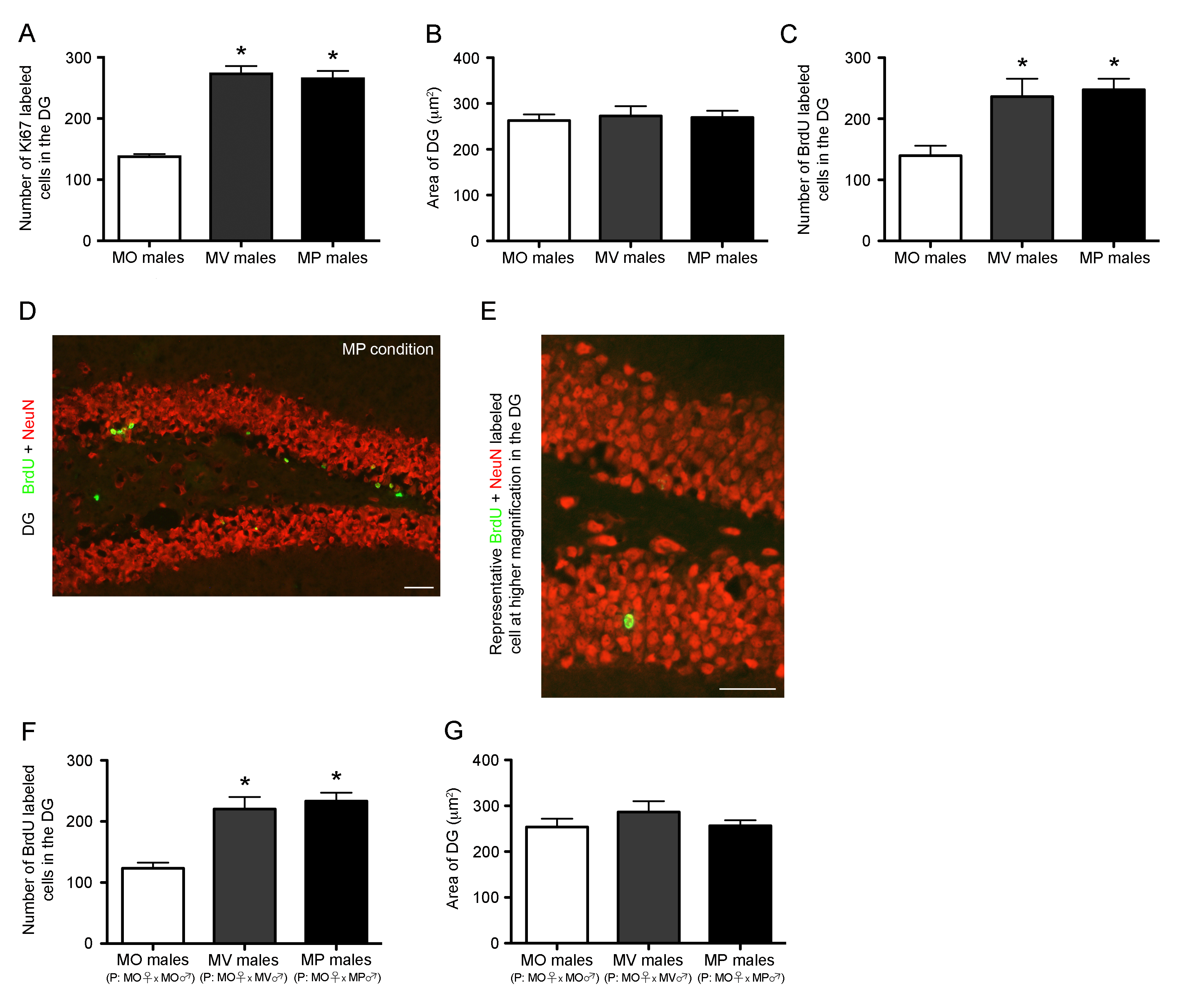

Supplement: Figure S4 — Enhanced parental care increases cell proliferation in the adult male dentate gyrus (DG). (A) Adult males raised in the maternal-virgin (MV) and maternal-paternal (MP) environments, have greater numbers of Ki67-labeled cells (mean±SEM) in the DG than adult males raised in a maternal-only (MO) environment. (B) The area (µm2) of the DG does not differ between MO, MV and MP males (mean±SEM). (C) Stereological quantification of BrdU-labeled cells in the DG revealed that MV and MP males have a greater number of BrdU-labeled cells than MO males (mean±SEM). (D) Representative fluorescent micrograph of BrdU-NeuN double-labeled cells in the DG of males raised in the maternal paternal environment. (E) Representative BrdU-NeuN double-labeled cells in the DG at higher magnification (40X). Bars in both D and E represent 50 µm. (F) Stereological analyses demonstrated that the number of BrdU-labeled cells in the DG is greater in MO males that are offspring of MV and MP fathers, compared to MO males that are offspring of MO fathers (mean±SEM). (G) The area (µm2) of the DG does not differ between MO males that are offspring of MO, MV, or MP fathers (mean±SEM). (TIF) [file pone.0062701.s004.tif]

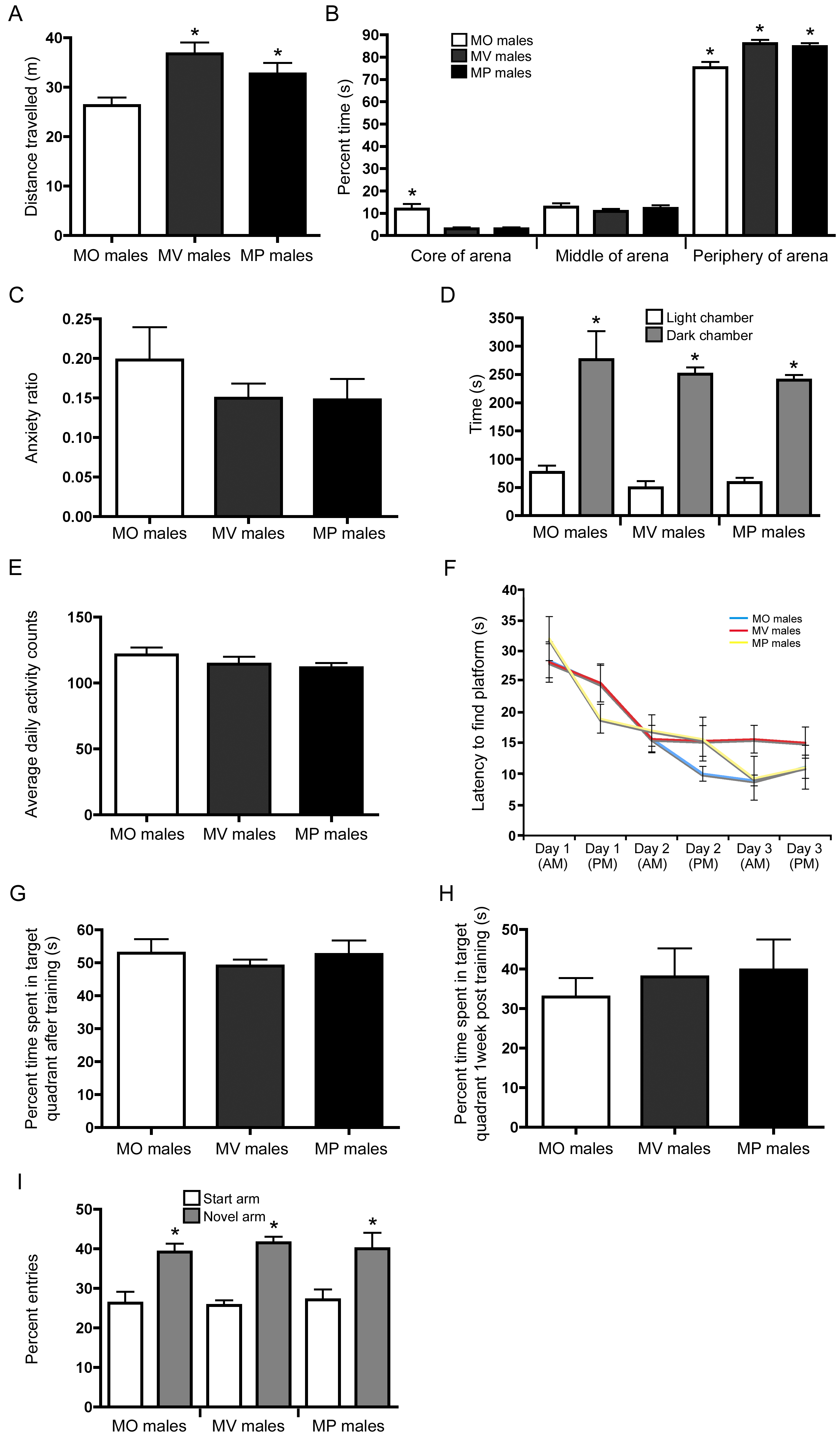

Supplement: Figure S5 — Assessment of general anxiety and spatial memory of adult males. (A) Males raised in maternal-virgin (MV) (n = 11) and maternal-paternal (MP) (n = 10) environments travel a greater distance in the open field compared to males raised in a maternal-only (MO) environment (n = 12) (mean±SEM). (B) MO males spend a greater percentage of time in the core of the arena compared to MV and MP males. However, MO, MV, and MP males, all spend a greater amount of time in the periphery of the open field (mean±SEM). (C) The general anxiety ratio of MO (n = 6), MV (n = 7), and MP (n = 7) males did not differ when measured using the elevated plus maze (mean±SEM). (D) When MO (n = 6), MV (n = 7), and MP (n = 7) males were placed in the light-dark choice task, they exhibited equal preference to spend more time in the dark-side of the chamber (mean±SEM). (E) MO, MV, and MP males show no difference in home cage activity (mean±SEM) (n = 6 for each group). (F) All males display equal ability to find a hidden platform in the Morris water maze during training (mean±SEM) (n = 8 for each group). (G and H) All males display equal time spent in the target quadrant where the hidden platform was previously placed when assessed one day after training, as well as seven days after training, respectively (mean±SEM). (I) The Y-maze was used to assess place recognition in MO (n = 6), MV (n = 7), and MP males (n = 7), which all showed equal levels of entry into the novel arm of the maze (mean±SEM). (TIF) [file pone.0062701.s005.tif]

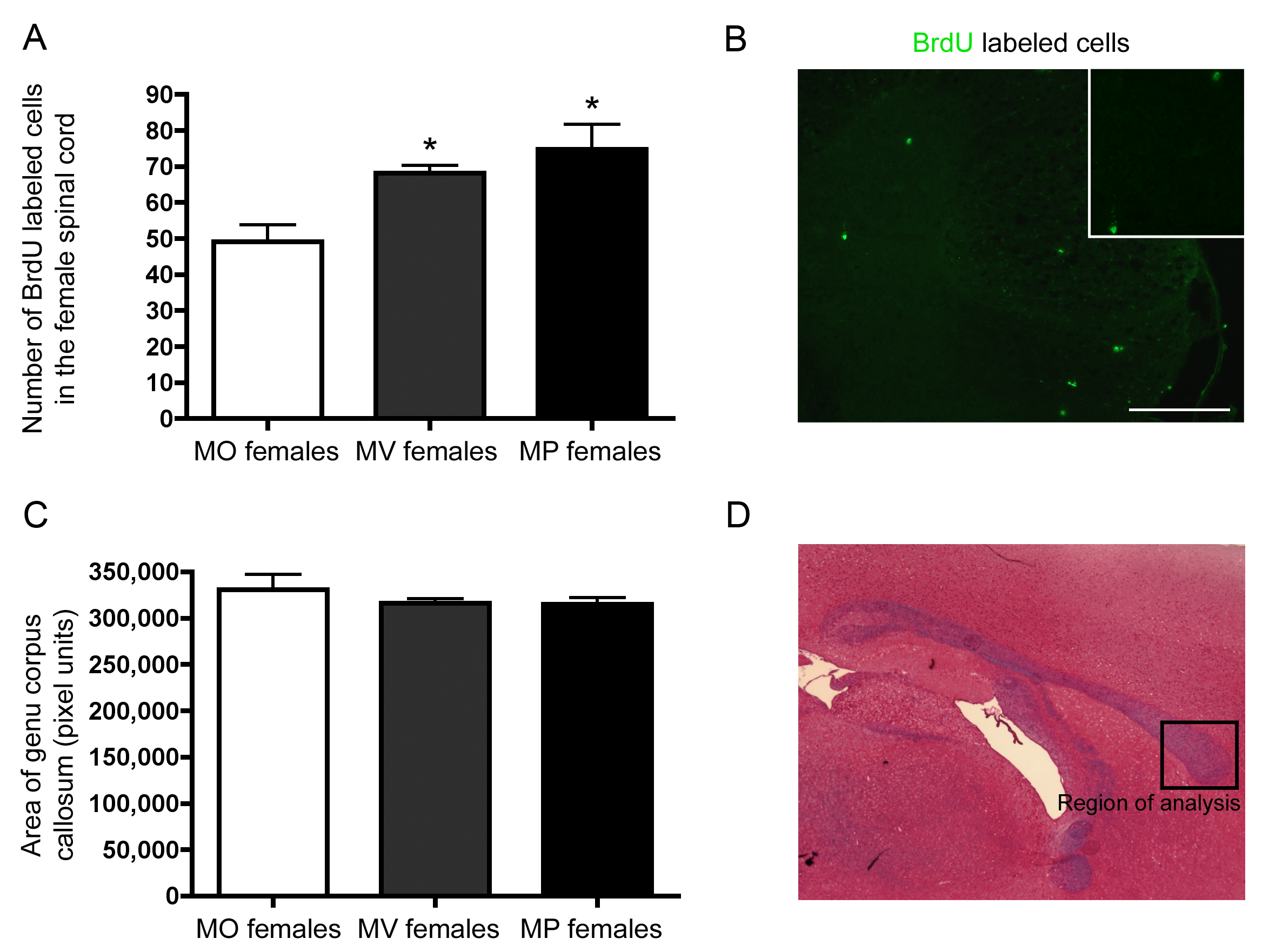

Supplement: Figure S6 — Enhanced parental care increases cell proliferation in the adult female spinal cord. Size of the adult corpus callosum is not affected in females raised in different parental care environments. (A) Adult females raised in maternal-virgin (MV) and maternal-paternal (MP) environments, have greater numbers of BrdU-labeled cells in the spinal cord (mean±SEM) than adult females raised in a maternal-only (MO) environment. (B) Fluorescent micrograph of BrdU-labeled cells in the spinal cord. Bar represents 50 µm. (C) The area of the adult genu of corpus callosum of MO, MV and MP females does not differ (mean±SEM) (n = 6 for each group). (D) Region of analysis depicted on a sagittal section stained with Eriochrome cyanine. (TIF) [file pone.0062701.s006.tif]

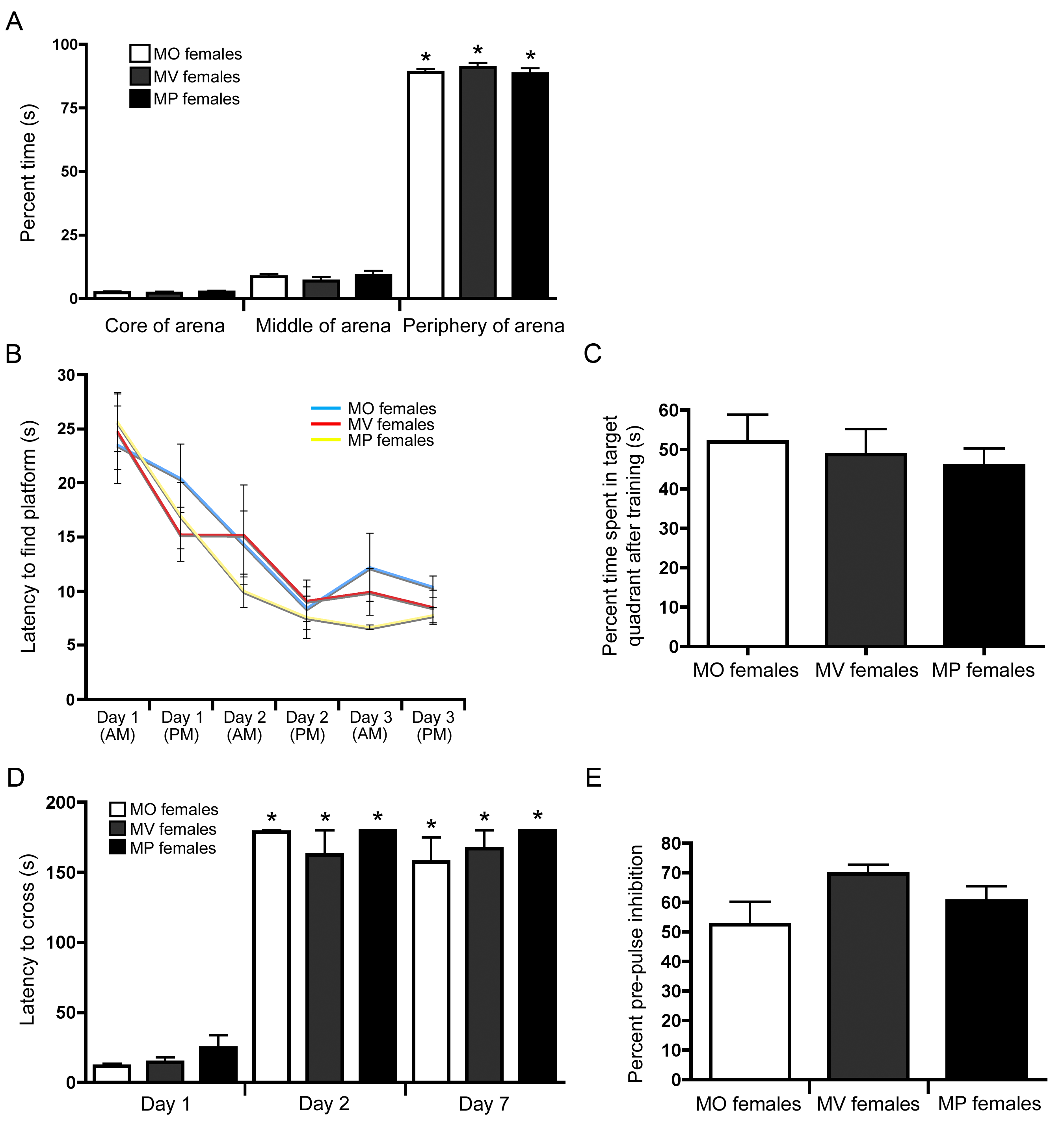

Supplement: Figure S7 — Assessment of general anxiety, spatial memory, and prefrontal cortex-dependent learning and memory in adult females. (A) In the open field, females raised in maternal-only (MO) (n = 8), maternal-virgin (MV) (n = 8), and maternal-paternal (MP) (n = 10) environments spent more time in the periphery than the core or middle of the arena (mean±SEM). (B) MO (n = 8), MV (n = 8), and MP (n = 10) females displayed equal ability to locate a hidden platform in the Morris water maze. (C) A probe trial conducted one day after training in the Morris water maze revealed no difference in the percentage of time MO, MV, and MP females spent in the quadrant where the hidden platform was previously located (mean±SEM). (D) MO (n = 8), MV (n = 8), and MP (n = 10) females equally demonstrate a memory to not cross to the novel side of the chamber in the passive-avoidance task the day after training (Day 2) and one week after training (Day 7) (mean±SEM). (E) MO (n = 8), MV (n = 8), and MP (n = 10) females show no difference in percent pre-pulse inhibition (mean±SEM). (TIF) [file pone.0062701.s007.tif]
